# Outstanding 1200 °C Oxidation Resistance in a Novel Multi‐Principal Element Alloy via Lattice Distortion‐Induced Diffusion Suppression

**DOI:** 10.1002/advs.202522526

**Published:** 2026-02-06

**Authors:** Xinyu Zhang, Weiyan Lv, Xinguang Wang, Chuanmin Jia, Yizhou Zhou, Keqiang Qiu, Jianqiang Wang

**Affiliations:** ^1^ Shenyang National Laboratory for Materials Science Institute of Metal Research, CAS Shenyang P. R. China; ^2^ School of Materials Science and Engineering Shenyang University of Technology Shenyang P.R. China; ^3^ Wangxuan Institute of Computer Technology Peking University Beijing P.R. China

**Keywords:** 1200 °C oxidation resistance, lattice distortion, multi‐principal element alloy, selective oxidation

## Abstract

The ongoing demand for high‐thrust turbine engines necessitates the advance of next‐generation structural materials capable of withstanding higher temperatures. Commercial MCrAlY alloy, used as bond coats crucial for thermal barrier coating (TBC) systems, face a fundamental temperature ceiling of ∼1100 °C due to accelerated oxidation and spallation. Here, we design a novel Y and Hf co‐doped NiCoCrAl‐type multi‐principal element alloy (MPEA) that achieves exceptional 1200 °C oxidation resistance primarily through lattice distortion‐induced diffusion suppression. Compared with typical NiCoCrAlY alloy, the MPEA exhibits 59% lower in thermally grown oxide (TGO) growth rate, as well as negligible TGO spallation after 500 h at 1200°C. This performance stems from a significantly refined eutectic structure enabling rapid formation of a protective Al_2_O_3_ scale during initial oxidation, coupled with lattice distortion that elevates vacancy formation energy and Al migration barriers within the Al‐depletion zone (ADZ), drastically reducing sustained diffusion rates. This co‐design strategy, integrating tailored microstructure and lattice distortion, establishes a new paradigm for ultra‐stable performance in extreme environments.

## Graphical abstract

The unique lamellar structure of the eutectic alloy promotes selective oxidation of Al initially, while lattice distortion‐induced diffusion suppression slows oxide growth during steady state. Combining these strategies, a Multi‐Principal Element Alloy with excellent oxidation resistance at 1200℃ was designed.

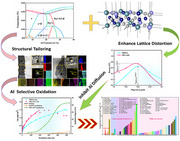

## 1 Introduction

Thermal barrier coatings (TBCs), extensively employed for thermal insulation and corrosion protection within aircraft engine hot sections, comprise a metallic bond coat, and a ceramic top coat [1, 2, 3, 4, 5]. The bond coat critically serves two functions: adhering the ceramic layer to the metallic substrate and protecting the substrate from oxidation. This protection depends on the bond coat's ability to form a continuous and dense alumina‐based thermally grown oxide (TGO) [6, 7, 8]. As aero‐engines development progresses toward higher thrust‐to‐weight ratios, turbine inlet temperature continues to rise [4, 9, 10]. However, conventional NiCoCrAlY (MCrAlY) bond coat alloys exhibit significantly accelerated oxidation rates above 1100°C. This rapid oxide scale growth leads to excessive TGO thickening. Crucially, once the accumulated elastic strain energy at the TGO/alloy interface surpasses the fracture toughness of the oxide and interface, catastrophic TGO spallation and coating failure occur [6, 11, 12]. Consequently, there is an urgent and unmet need to develop bond coats capable of maintaining superior oxidation resistance and TGO stability at temperatures approaching 1200°C for next‐generation turbine engines.

Significant efforts have been made to enhance the high‐temperature oxidation resistance of MCrAlY alloys, employing strategies such as reactive element (RE) additions [13, 14], grain refinement [15, 16, 17], platinum alloying [17, 18], gradient coatings [19, 20, 21] and pre‐oxidation treatments [17, 22, 23, 24]. While these approaches can yield marginal improvements, they consistently fall short of achieving the required performance at 1200°C. A fundamental limitation lies in their inability to simultaneously address the two critical, sequential stages of the oxidation process: 1) rapid formation of an exclusive Al_2_O_3_ scale during the initial transient stage, and 2) suppression of TGO growth kinetics during prolonged steady‐state oxidation. Alternative material systems, including AlCoCrFeNi high‐entropy alloys (HEAs) and β‐NiAl intermetallics, have also been explored as potential bond coat replacements [6, 25, 26, 27, 28, 29]. However, their high Fe or Al content often accelerates detrimental interdiffusion with single‐crystal superalloy substrates, promoting the formation of brittle topologically close‐packed (TCP) phases within secondary reaction zones (SRZs) that severely compromise the substrate's service life [30, 31, 32, 33].

Achieving durable oxidation resistance requires success in two sequential stages: [15] Initial rapid establishment of a continuous Al_2_O_3_ scale drastically reduces oxygen partial pressure at the alloy/TGO interface, blocking the formation of non‐protective oxides (e.g., porous spinel) [34, 35]. Crucially, the presence of discontinuous spinel domains compromises TGO continuity and its barrier function, accelerating elemental diffusion during prolonged exposure [17, 36, 37]. Within the selective oxidation framework [38]. eutectic alloys with their inherent lamellar microstructures featuring elongated phases, are promising as they can optimize Al supply pathways during the initial oxidation, effectively lowering the critical Al concentration needed for exclusive Al_2_O_3_ scale formation [39, 40, 41]. Subsequent long‐term protection hinges on suppressing elemental diffusion through the Al‐depletion zone (ADZ) during steady‐state growth [42, 43]. While eutectic microstructures can enhance initial Al supply—they alone cannot ensure long‐term stability. Here, the inherent entropy effects of multi‐principal element alloys (MPEAs) offer a transformative advantage [40, 44, 45, 46, 47]. The severe lattice distortion generated by high configurational entropy significantly increases vacancy formation energies and cation migration barriers [48, 49, 50]. This intrinsic entropy‐driven diffusion suppression mechanism is critical for retarding steady‐state scale growth kinetics, the key limitation of conventional strategies at extreme temperatures. Building upon these two mechanisms, eutectic MPEA alloys demonstrate the potential to simultaneously optimize oxidation behavior across both initial and long‐term stages. Since their initial report by Lu et al. [51], eutectic MPEAs have been extensively validated by numerous researchers for their exceptional properties, including corrosion resistance [52, 53], mechanical performance [54, 55, 56, 57, 58, 59], and radiation tolerance [60, 61, 62]. In particular, the NiCoCrAl‐type eutectic alloy system, proposed and mechanistically substantiated by Liu et al. [57], exhibits remarkable mechanical properties. This provides additional performance assurance—beyond oxidation resistance—for its application as a bond coat material in thermal barrier coating systems.

To overcome the limitations of existing strategies and leverage the unique advantages of entropy modulation, this study proposes a novel dual‐strategy approach targeting both critical oxidation stages: 1) Utilizing phase diagram thermodynamics, we determine an eutectic Al content to achieve a lamellar microstructure. This structure is designed to enhance Al supply during initial oxidation, promoting the rapid formation of a continuous, exclusive Al_2_O_3_ scale by effectively lowering the critical Al concentration. 2) Elevating Co and Cr concentrations toward an equiatomic ratio with Ni maximizes configurational entropy and the resulting severe lattice distortion within the ADZ formed beneath the TGO. This entropy‐driven lattice distortion is engineered to increase vacancy formation energy and Al migration barriers, thereby reducing sustained Al diffusion rates to the growing scale. The resulting Ni_32_Co_25_Cr_25_Al_18_ MPEA demonstrates exceptional oxidation resistance and drastically reduced TGO spallation at 1200°C, significantly outperforming commercial MCrAlY. Selective oxidation analysis and first‐principles calculations elucidate the underlying performance enhancement mechanisms, providing fundamental scientific guidance for designing next‐generation ultra‐high‐temperature bond coat materials.

## 2 Results

**FIGURE 1:**
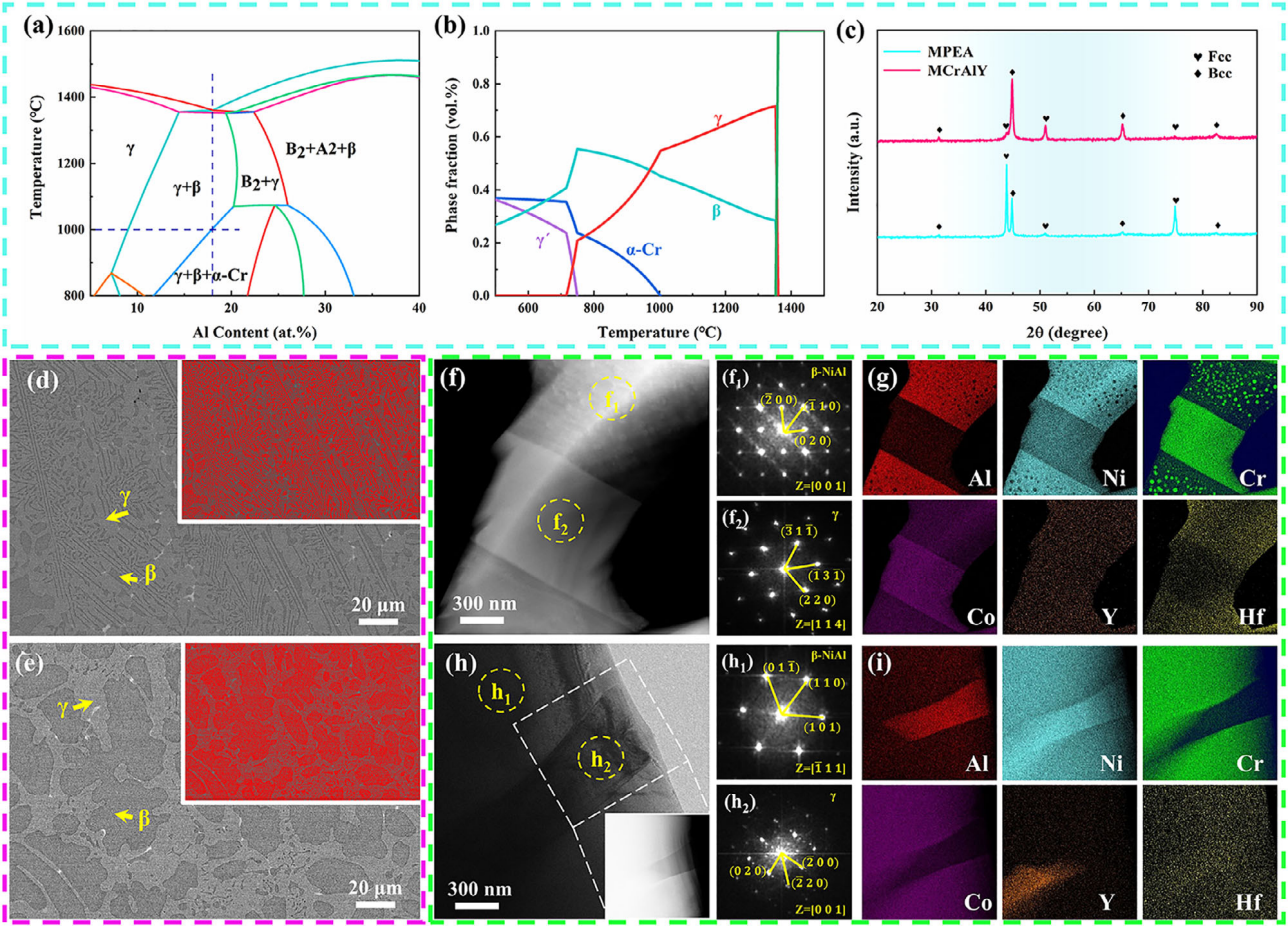
Phase diagram calculation, phase analysis, and microstructural characterization of the MPEA and MCrAlY. (a): Pseudo‐binary phase diagram of Ni_50‐x_Co_25_Cr_25_Al_x_ system calculated using the CALPHAD method; (b): Phase fraction of the designed alloy (Ni_32_Co_25_Cr_25_Al_18_) calculated using the CALPHAD method; (c): XRD patterns of the MPEA and MCrAlY; (d, e): SEM images display a lamellar microstructure in MPEA, distinct from typical ellipsoidal microstructure in MCrAlY (inset enhanced contrast for clarity); (f): TEM image of MPEA; f_1,_ f_2_): FFT of the region labeled f_1_ and f_2_ in f; (h): TEM image of MCrAlY; h_1,_ h_2_): FFT of the region labeled h_1_ and h_2_ in h; (g): STEM‐EDS maps of (f) reveal the existence of Cr‐rich nanoprecipitates in β‐phase; (i): STEM‐EDS maps of the inset region in (h) reveal the absence of Cr‐rich nanoprecipitates within the β‐phase of the MCrAlY.

The Multi‐Principal Element alloy (MPEA) of the Ni_32_Co_25_Cr_25_Al_18_ is designed in terms of the CALPHAD method. The calculated pseudo‐binary phase diagram of the Ni_50‐x_Co_25_Cr_25_Al_x_ system (Figure 1a) shows that a eutectic structure forms at 18 at.% Al, which agrees with prior literature [57] and confirms the accuracy of our calculation. Phase fraction calculations for the (Ni_32_Co_25_Cr_25_Al_18_) MPEA (Figure 1b) indicate the formation of a dual‐phase FCC/BCC eutectic microstructure during solidification. Additionally, α‐Cr phase precipitation is predicted below ∼1000°C. To validate the CALPHAD predictions for the designed MPEA, both MPEA and MCrAlY with identical Y and Hf additions were fabricated via arc melting and subsequently characterized. X‐ray diffraction (XRD) patterns (Figure 1c) confirm dual‐phase γ/β structures in both alloys, consistent with Figure 1a,b. Scanning Electron Microscopy (SEM) images (Figure 1d,e) reveal a continuous light gray matrix with dark gray phases. The MPEA displays characteristic lamellar eutectic microstructure (Figure 1d), while MCrAlY exhibits ellipsoidal morphology. Transmission Electron Microscopy (TEM) analysis of MPEA and MCrAlY (Figure 1f,h) confirms γ and β‐NiAl phases via High‐Angle Annular Dark Field (HAADF) imaging and Fast Fourier Transform (FFT) patterns. Scanning Transmission Electron Microscopy Energy Dispersive X‐ray Spectroscopy (STEM‐EDS) mapping (Figure 1g,i) shows Co/Cr/Ni enrichment in γ‐phase and Ni/Al enrichment in β‐phase. In addition, comparative analysis of Figure 1g,i reveals nanoscale Cr‐rich precipitates within the β‐phase of MPEA, a feature absent in MCrAlY. These Cr‐rich nanoprecipitates are indexed as α‐Cr phase sharing the BCC structure with the β‐matrix [63, 64], rationalizing the absence of superlattice reflections in Figure 1f_1_. Their formation attributed to Cr content exceeding β‐phase solubility.

To elucidate the differential α‐Cr precipitation behavior, we calculated the pseudo‐binary phase diagram for the Ni_48‐x_Co_23_Cr_17_Al_x_ (wt.%) system and phase fraction diagram for MCrAlY (Figure S1). These reveal a narrow γ+β+α‐Cr phase field and lower α‐Cr solidus temperature in MCrAlY, indicating suppressed precipitation relative to MPEA. Geometric phase analysis (GPA) of β‐phase and γ‐phase High‐Resolution TEM (HRTEM) images further quantified atomic‐scale strain distributions (*ε_xx_*), as shown in Figure S2. Areal fractions of compressive (yellow) and tensile (blue) strain regions were extracted via ImageJ (Figure S2). Quantitative analysis shows MPEA's β‐phase and γ‐phase contain 68.5% and 10.7% larger high‐strain areas, respectively, than MCrAlY, confirming enhanced lattice distortion in MPEA.

**FIGURE 2:**
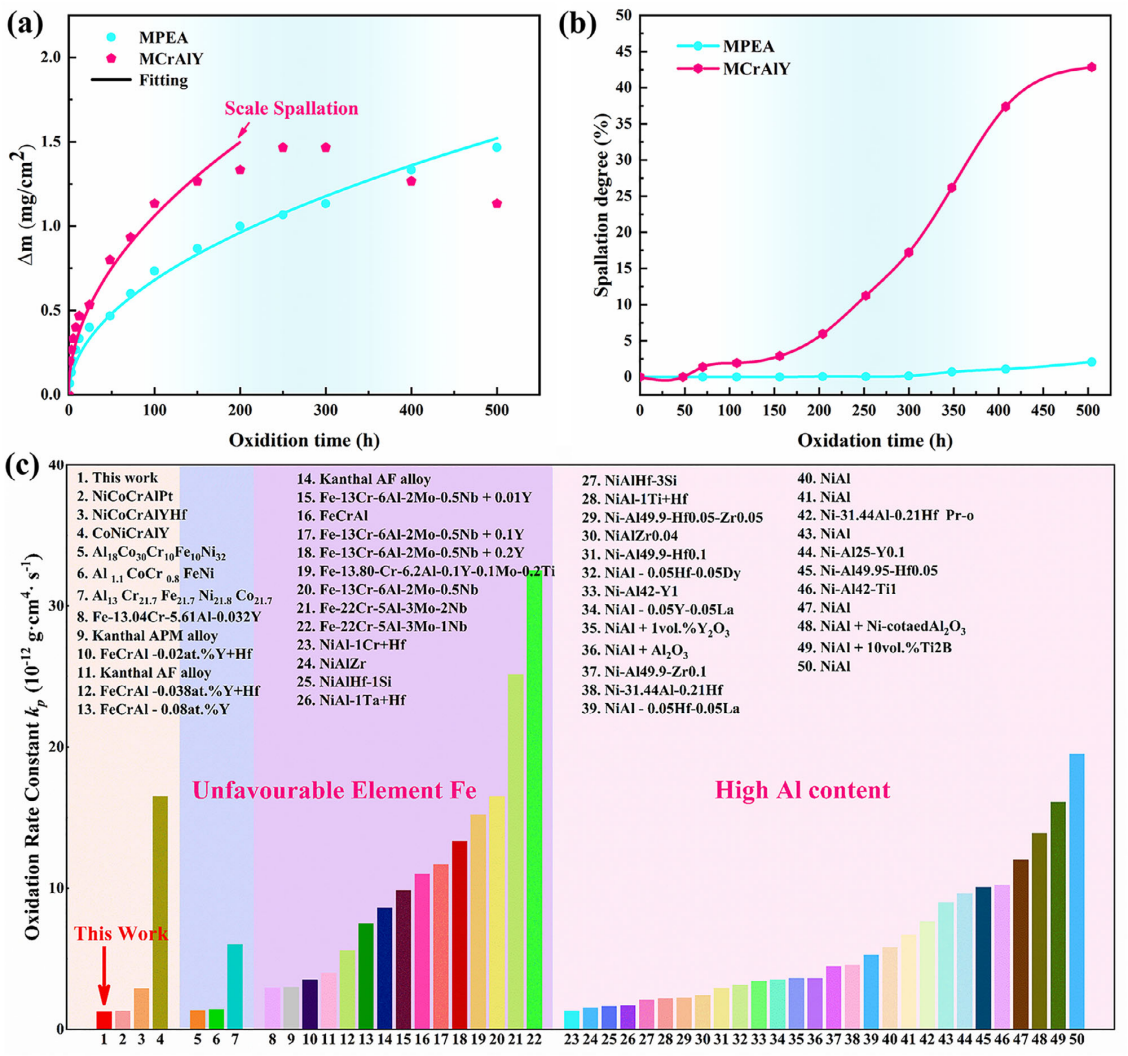
Analysis of oxidation resistance at 1200 °C of the MPEA and MCrAlY. (a): Isothermal oxidation kinetics curves of MPEA and MCrAlY at 1200°C, exhibiting slower oxide scale growth rate in MPEA; (b): Time‐dependent progression of oxide spallation area fraction during 1200 °C cyclic oxidation, demonstrating superior oxides adhesion in MPEA compared to MCrAlY; (c): Comparison of oxidation rate constant (*k_p_*) between the MPEA and other Al_2_O_3_‐forming alloys, indicating significant oxidation resistance advantages of the present alloy. The detailed data can be found in Table S1 (Supporting Information).

To evaluate oxidation resistance, isothermal and cyclic oxidation tests were conducted on MPEA and MCrAlY at 1200°C. Figure 2a,b presents oxidation kinetics (500 h isothermal) and spallation behavior (cyclic). MCrAlY exhibits linear mass gain (*Δm*) kinetics before 250 h, followed by *Δm* reduction indicating severe oxide scale spallation. In contrast, MPEA shows significantly lower *Δm* with continuous parabolic growth kinetics throughout testing, confirming superior oxidation resistance without notable spallation. Both alloys follow approximately parabolic oxidation kinetics. According to Wagner's theory, the oxidation rate constant is derived from *Δm* data [65].

(1) $$\Delta m^{2}=k_{p}\;t$$

where *k_p_* and *t* stand for the oxidation rate constant and oxidation time, respectively. The calculated oxidation rate constant of MPEA is 1.28 × 10^−12^ g^2^ cm^−4^ s^−1^, which is 58% lower than that (3.11 × 10^−12^ g^2^ cm^−4^ s^−1^) of the MCrAlY.

To comprehensively assess oxidation resistance, cyclic oxidation spallation behavior of MPEA and MCrAlY was quantified. As shown in Figures 2b and S3, MCrAlY exhibits severe oxide scale degradation: spallation initiates after 70 h at 1200°C, rapidly progresses to 15% area loss by 300 h (indicating protective failure), and exceeds 40% after 500 h. Conversely, MPEA shows negligible spallation (<2%) throughout 500 h testing. This demonstrates MPEA's markedly superior spallation resistance compared to MCrAlY.

To highlight the excellent oxidation resistance of the MPEA at 1200°C, its oxidation rate constant (*k_p_*) is compared against other Al_2_O_3_‐forming alloys (AlCoCrFeNi, FeCrAl, and NiAl) in Figure 2c (The detailed data can be found in Table S1. Among these alloys, the oxidation rate of MPEA is substantially comparable to those of NiCoCrAlPt (1.3 × 10^−12^ g^2^ cm^−4^ s^−1^), AlCoCrFeNi (1.33 × 10^−12^ g^2^ cm^−4^ s^−1^), and NiAlHf (1.3 × 10^−12^ g^2^ cm^−4^ s^−1^), all of which exhibit extremely low oxidation rates. However, the extremely high Pt content in NiCoCrAlPt renders it extremely expensive for commercialization. Similarly, the high Fe content in AlCoCrFeNi and the high Al content in NiAlHf significantly promote the risk of severe interdiffusion with single‐crystal substrates when employed as bond coats [31, 32]. Furthermore, the MPEA developed in this work displays the lowest oxidation rate, endowing its exceptional oxidation resistance. In summary, this positions MPEA as a superior bond coat candidate for next‐generation thermal barrier coating systems.

Next‐generation thermal barrier coating bond coats require not only exceptional oxidation resistance but also adequate mechanical properties. To evaluate this critical aspect, we compared the tensile performance of both alloys at room temperature. As shown in Figure S4, the commercial NiCoCrAlY alloy exhibits an ultimate tensile strength of 635 MPa with an elongation of 1.1%, while our MPEA demonstrates significantly superior properties with a strength of 711 MPa and an elongation of 2.7%. The simultaneous enhancement of both strength and ductility observed in our MPEA originates from its unique eutectic multi‐principal element alloy architecture, characterized by finely distributed and intimately alternating soft and hard phases—a structural advantage well documented in previous studies [51, 55, 57]. This mechanical superiority further highlights the potential of our MPEA as a high‐performance replacement for conventional NiCoCrAlY bond coats.

**FIGURE 3:**
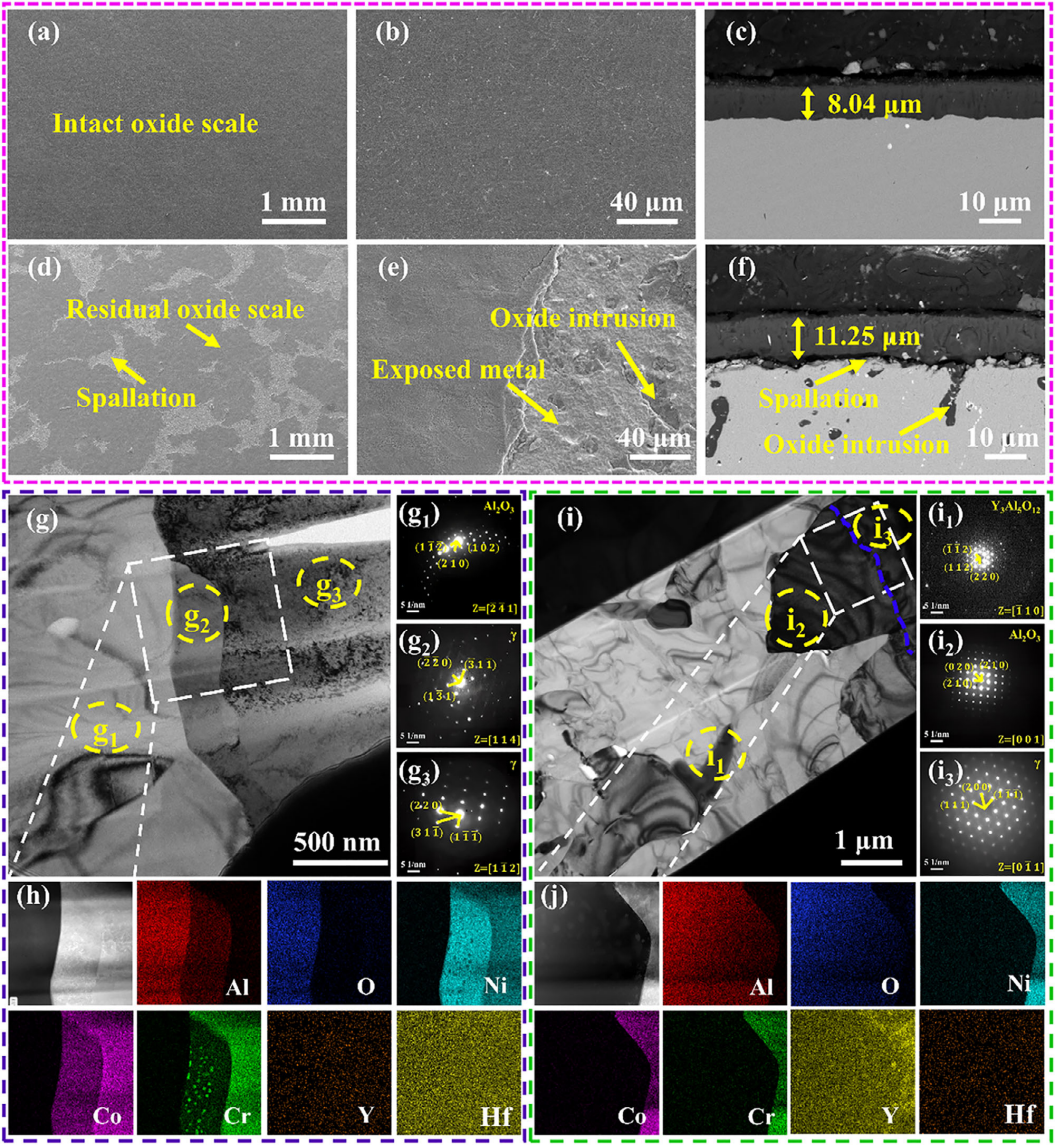
Characterization of oxide scale after 500 h at 1200°C. (a‐c): SEM images of the surface (a, b) and cross‐sectional (c) of MPEA oxide scale display an intact oxide scale and the oxide scale thickness ∼8.04 µm; (d‐f): SEM images of the surface and cross‐sectional of MCrAlY oxide scale show severe spallation and oxide intrusion with a oxide scale thickness ∼11.25 µm; (g): TEM image of oxide scale‐substrate interface for MPEA; g_1_‐g_3_): SAED patterns of the region g_1_ (Al_2_O_3_), g_2_ (γ‐phase), and g_3_ (γ‐phase); (h): STEM image of the white‐cubic region in (g) and corresponding elemental mapping reveal the distribution of α‐Cr in region g_2_; i): TEM image of oxide scale‐substrate interface for MCrAlY; i_1_‐i_3_): SAED patterns of the region i_1_ (Y_3_Al_5_O_12_), i_2_ (Al_2_O_3_), and i_3_ (γ‐phase); (j): STEM image of the white‐cubic region in (i) and corresponding elemental mapping.

To elucidate the oxidation resistance mechanisms of MPEA, oxide scale surfaces and cross‐sections were characterized via SEM and TEM (Figure 3). After 500 h at 1200°C, MPEA maintains a spallation‐free, continuous oxide scale (Figure 3a,b). Cross‐sectional analysis (Figure 3c) reveals an adherent 8.04 µm‐thick Al_2_O_3_ scale (confirmed by EDS mapping, Figure S5) with no defects and interfacial delamination, indicating exceptional scale‐substrate adhesion. In contrast, MCrAlY exhibits severe scale spallation at the scale‐substrate surface after identical exposure (Figure 3d,e), with exposed substrate showing oxide intrusions. Cross‐sectional microstructural analysis reveals an 11.25 µm‐thick oxide scale on MCrAlY, exhibiting great interfacial delamination and internal oxidation zone (Figure 3f). The thinner oxide scale on MPEA (8.04 vs. 11.25 µm) and superior interfacial integrity directly correlate with its lower oxidation kinetics and enhanced spallation resistance observed in Figure 2a.

More detailed characterization of the scale‐substrate interface by TEM is presented in Figures 3g,i. 3g shows the scale‐substrate interface of MPEA, where the region labeled g_1_ is identified as Al_2_O_3_ by SAED (Figure 3g_1_), and g_3_ as γ‐phase (Figure 3g_3_). A distinct interfacial zone (g_2_) features uniformly dispersed Cr‐rich nanoprecipitates (Figure 3g_2_). SAED confirms the γ‐phase matrix in this region (Figure 3g_2_), while correlation with prior TEM results (Figure 1f) definitively indexes these precipitates as α‐Cr. The scale‐substrate interface of MPEA characterized by STEM is shown in Figure 3i. Regions labeled i_1_, i_2_, and i_3_ are identified by SAED as Y_3_Al_5_O_12_ (Figure 3i_1_), Al_2_O_3_ (Figure 3i_2_), and γ‐phase matrix (Figure 3i_3_), respectively. In contrast to MPEA, no α‐Cr is detected at the scale‐substrate interface of MCrAlY.

Reactive elements Y and Hf are critical for enhancing the oxidation resistance of alloys, and investigating their role in oxidation behavior is essential. Using STEM‐EDS, we analyzed the elemental distribution within Al_2_O_3_ grains and grain boundaries in the MPEA and MCrAlY alloys, as shown in Figure S6. According to Figures S6a_3_, S6a_4_, S6b_3_, and S6b_4_, both Y and Hf are enriched at the Al_2_O_3_ grain boundaries. This observation aligns with findings reported by Liu et al. [66], where Y and Hf enrichment at Al_2_O_3_ grain boundaries effectively blocks outward Al diffusion through the oxide scale, thereby reducing the growth rate of Al_2_O_3_. Additionally, studies have shown that reactive elements Y and Hf can also pin interfaces, increase the work of separation, reduce interfacial energy, and adsorb impurities such as S, all contributing to enhanced oxide scale adhesion and reduced oxidation rates [66, 67, 68, 69]. However, in this study, the addition levels of reactive elements are identical in both alloys. Therefore, the reactive elements are not the factor responsible for the differences in oxidation performance between the two alloys.

It is recognized that oxide scale spallation occurs when the elastic strain energy *G* accumulated within the scale exceeds the interfacial toughness. The elastic strain energy *G* is given by: [17, 70]

(2) $$G=\frac{\left(1-v_{ox}^{2}\right)}{2E_{ox}}\;\sigma^{2}h_{ox}$$

where *h_ox_* is the thickness of the Al_2_O_3_ scale, *v_ox_* and *E_ox_* are the Poisson’ ratio and Young’ modulus of the Al_2_O_3_ scale, respectively. *σ* is the residual stress in the Al_2_O_3_ scale. According to Equation 2, the elastic strain energy *G* increases with both Al_2_O_3_ scale thickness *h_ox_* and residual stress *σ* within the Al_2_O_3_ scale. The slower growth kinetics and reduced Al_2_O_3_ scale thickness in the MPEA have been illustrated in Figure 2a. Therefore, assessing residual stresses within the Al_2_O_3_ scale is critical for elucidating its spallation resistance mechanisms at elevated temperatures. The residual stress *σ* can be measured using photoluminescence piezospectroscopy (PLPS) technique, by: [17]

**FIGURE 4:**
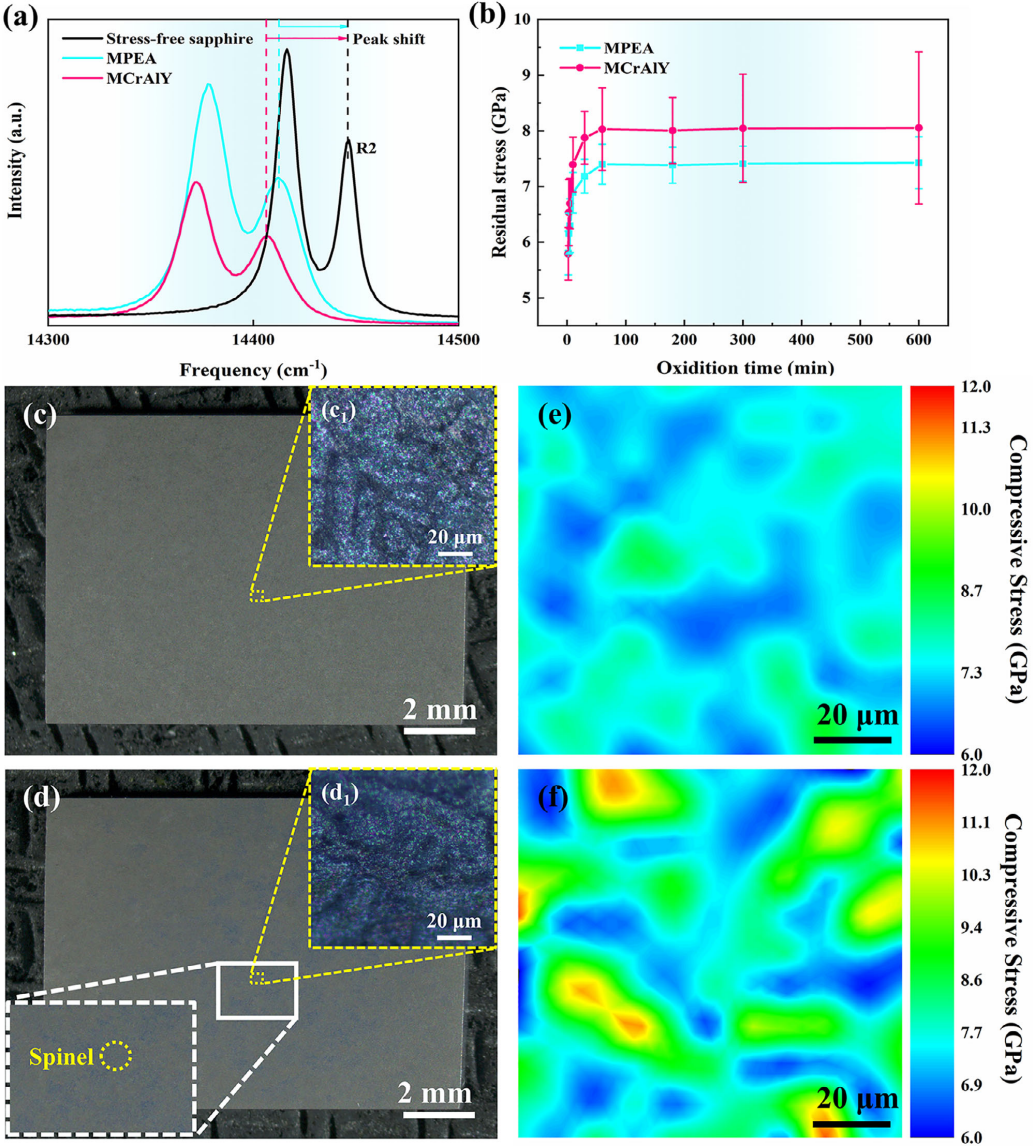
Analysis of residual stress within oxide scale. (a): Peak shift of R2 line collected from the Al_2_O_3_ scale and the stress‐free sapphire was utilized to be as stress reference; (b): Time evolution of residual stresses in Al_2_O_3_ scales for MPEA and MCrAlY during initial oxidation stage (<10h) (The average value is obtained by 121 measurement points and the error bar denote standard deviation); (c): Stereomicroscopic image of the Al_2_O_3_ scale surface on MPEA After 10 h oxidation at 1200°C; c_1_): Enlarged image of the yellow‐cubic region in (c) using confocal Raman microprobe microscope; (d): Stereomicroscopic image of the Al_2_O_3_ scale surface on MCrAlY After 10 h oxidation at 1200°C, inset: Enlarged image of the white‐cubic region in (d) reveal the formation of blue oxide; d_1_): Enlarged image of the yellow‐framed region in (d) using confocal Raman microprobe microscope; (e, f): Residual stress distribution maps within (c1), (c2) depicting low residual stress and homogeneous residual stress distribution within the Al_2_O_3_ scale of MPEA.

(3) σ=Δv/5.07

where *Δv* represents the R2 peak shift between the Al_2_O_3_ scale and stress‐free sapphire reference, with the peak shift schematic illustrated in Figure 4a. Figure 4b shows the evolution of residual stresses in Al_2_O_3_ scale during the first 10 h of oxidation. Stress values represent the average of 121 measurement points across a 100 × 100 µm grid. Error bars denote standard deviation. Both alloys exhibit rapid stress accumulation during initial oxidation (<1 h), stabilizing after ∼1 h. Crucially, MCrAlY develops significantly higher compressive stresses (∼ ‐8.0 GPa) than MPEA (∼ ‐7.4 GPa).

Figure 4c,d present the macroscopic surface morphologies of Al_2_O_3_ scales on MPEA and MCrAlY after 10 h oxidation at 1200°C. Unlike MPEA, MCrAlY exhibits blue‐colored spinel oxides (e.g., (Ni, Co) Cr_2_O_4_) on its scale surface (Figure 4d inset). To evaluate the impact of spinel formation on spallation resistance, residual stress distribution maps were generated within representative areas demarcated in Figure 4c,d (yellow boxes), as shown in Figure 4e,f. MCrAlY exhibits significantly larger stress concentration zones in its Al_2_O_3_ scale compared to the MPEA. These high‐stress regions preferentially serve as crack initiation sites, accelerating scale spallation. Conversely, MPEA's lower residual stresses (∼ ‐7.4 GPa avg.) and homogeneous stress distribution directly account for its superior spallation resistance.

## 3 Discussion

As established, our designed MPEA demonstrates exceptional oxidation resistance at 1200°C, characterized by retarded Al_2_O_3_ scale growth kinetics and extended spallation lifetime. Benchmarking against literature data further confirms MPEA's superior potential for thermal barrier coating bond‐coat applications. Subsequent mechanistic investigations will elucidate two critical design aspects:

### 3.1 Protective Al_2_O_3_ Scale Evolution During Initial Oxidation

**FIGURE 5:**
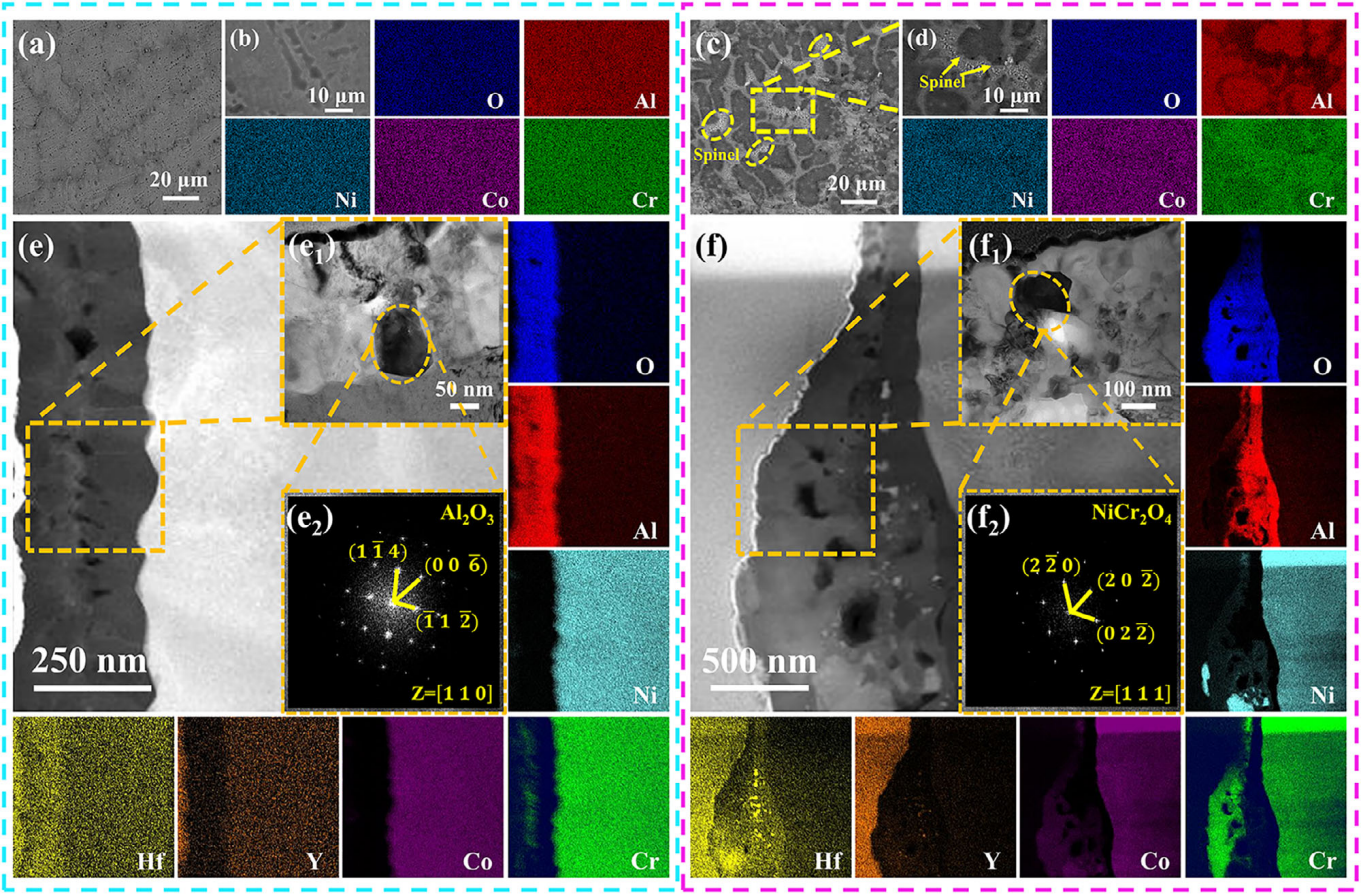
Characterization of oxide scales formed during the initial oxidation stage (3 min). (a): SEM image of MPEA's oxide scale surface reveals no formation of porous spinel; (b): Magnified SEM image and corresponding elemental mapping demonstrating exclusive Al_2_O_3_ composition of MPEA's oxide scale; (c): SEM image of MCrAlY's oxide scale surface reveals porous spinel formation specifically on γ‐phase surface; (d): Enlarged SEM image of the yellow‐cubic region in (c) and corresponding elemental mapping revealing dual‐phase composition of MCrAlY's oxide scale: Al_2_O_3_ and Cr‐rich porous oxides; (e): Cross‐sectional STEM image and corresponding elemental mapping of MPEA's oxide scale revealing comparable scale thickness on β‐phase and γ‐phase with predominantly Al_2_O_3_ composition (The selected sampling positions incorporated oxide scale formation on both β‐phase and γ‐phase surfaces. Due to Al depletion induced by oxidation, only the γ‐phase remained observable in the analyzed zone.); e_1_, e_2_): Enlarged STEM image of the orange‐cubic region in (e) and FFT pattern of orange‐cubic region in (e_1_) confirms the oxide as Al_2_O_3_; (f): Cross‐sectional STEM image and corresponding elemental mapping of MCrAlY's oxide scale reveal a duplex structure on γ‐phase surfaces: an outer Ni, Co and Cr‐enriched oxide layer overlying an inner Al_2_O_3_ base, with significantly greater thickness versus that of β‐phase surface; f1, f2): Enlarged STEM image of the orange‐cubic region in (f) and FFT pattern of orange‐cubic region in (f_1_) confirms the Ni, Co and Cr‐enriched oxide as NiCr_2_O_3_ spinel.

To unveil the initial oxidation processes of MPEA and MCrAlY, SEM and TEM were employed to characterize the oxide scales formed on both alloy specimens after 3 min oxidation at 1200°C. It is revealed that dense oxide scales formed uniformly on both β and γ‐phases of MPEA (Figure 5a,b). In contrast, porous oxides are observed on the γ‐phase of MCrAlY (Figure 1c), with EDS confirming their Cr‐rich composition (Figure 5d). To enable precise characterization of oxide, TEM specimens were prepared using a focused ion beam (FIB) system at the locations indicated in Figure S7. As shown in Figure 5e, Cross‐sectional HAADF imaging depicts that oxide layers formed on both β‐phase and γ‐phase surfaces of MPEA exhibit comparable thicknesses of approximately 270 nm. Integrated analysis of FFT pattern (Figure 5e_2_), and STEM‐EDS confirmed the oxide scales primarily consist of Al_2_O_3_ with only minute quantities of Cr_2_O_3_, demonstrating successful achievement of the exclusive growth of Al_2_O_3_. In contrast to MPEA, the oxide scale formed on the γ‐phase surface of MCrAlY presents a thickness of 1100 nm, remarkably larger than the 310 nm‐thick oxide scale developed on its β‐phase surface (Figure 5f). The STEM‐EDS result displays a duplex oxide scale structure formed on the γ‐phase surface of the MCrAlY alloy. The inner layer comprises an Al‐enriched oxide identified as Al_2_O_3_, while the outer layer exhibits Ni, Co, and Cr enrichment. FFT analysis confirms this Ni, Co, and Cr‐rich oxide corresponds to (NiCo)Cr_2_O_4_ with a characteristic spinel structure (Figure 5f_2_).

Theoretically, Al_2_O_3_ preferentially forms on Al‐rich β‐phase surfaces, while spinel phases predominantly develop on Al‐poor γ‐phase surfaces—consistent with our observations in Figure 5c,f. According to Wang's model, the exclusive growth of Al_2_O_3_ on γ‐phase surfaces requires sufficient Al flux to the interface to counter depletion, expressed mathematically as: [17, 38]

(4) $$N_{Al}^{\gamma }>N_{Al\left(min\right)}^{\gamma }\xi \left(z\right)$$

With

(5) $$N_{Al\left(min\right)}^{\gamma }=\frac{V_{m}}{V_{Al2O3}}\;{\left(\frac{\pi k_{p}}{2D_{Al}^{\gamma }}\right)}^{\frac{1}{2}}$$

where *Nγ Al* is the Al concentration of γ phase, *Nγ Al_(min)_* is the critical Al concentration, *V_m_* (7.2 cm^3^/mol) and *V_Al2O3_* (25.6 cm^3^/mol) are the molar volumes of the alloy and the Al_2_O_3_, respectively [17]; *Dγ Al* is the diffusion coefficient of Al in the γ‐phase at 1200°C, is calculated as 4.5 × 10^−12^ mm^2^/s using Thermo‐Calc software (Figure S8a. A linear regression was performed on Figure S8a, and the obtained fitting equation is shown in Figure S8b); and *k_p_* is the oxidation rate constant of MPEA and MCrAlY are calculated to be 8.90 × 10^−13^ and 3.56 × 10^−12^ cm^2^s^−1^, respectively. Substituting with required values into the Equation 5, the critical Al concentration of MPEA and MCrAlY are calculated as 9.8 at.% and 15.4 at.% respectively. EDS measurements of Al concentrations in the γ‐phase of MPEA and MCrAlY are presented in Table S2. Clearly, the γ‐phase Al content in MPEA (13.0 at.%) exceeds its critical Al concentration, while that in MCrAlY (11.8 at.%) falls below its critical Al concentration. Consequently, only the surface of γ‐phase of MCrAlY develops spinel oxides, validating our Figure 5 observations.

It should be noted that the critical aluminum concentration required for exclusive Al_2_O_3_ scale formation is also governed by the size and shape of β‐phase. In Equation 4, *ξ(z)* represents a parameter related to the size and shape of β‐phase, expressed as [38].

(6) $$\xi \left(z\right)={\left[1+\sum_{n=1}^{\infty }z\;exp\left(-\frac{z^{2}}{{\left(4n\right)}^{2}}\right)\int_{z/4n^{2}}^{\infty }exp\left(-n^{2}y^{2}\right)dy\right]}^{-1}\;$$

With

(7) $$z=P_{L}\;\sqrt{D_{Al}t}$$

Equation 6 can be reformulated using the complementary error function (Erfc) as follows:

(8) $$\xi \left(z\right)={\left[1+\frac{\sqrt{\pi }}{2}z\sum_{n=1}^{\infty }\frac{1}{n}\;exp\left(-\frac{z^{2}}{16n^{2}}\right)Erfc\left(\frac{z}{4n}\right)\right]}^{-1}\;$$

where *y* is an auxiliary variable, n is a positive integer. *D_Al_* is the interdiffusion coefficient of the alloy. *P_L_* is the number of intercepts by the β‐phase features in the alloy per unit length of a linear probe, and depends on the size, shape, and volume fraction of the β‐phase. According to Equation 8, *ξ(z)* decreases with increasing *P_L_*, indicating that alloys with lower *P_L_* values exhibit enhanced capability for exclusive Al_2_O_3_ formation. *P_L_* is defined by: [38]

(9) $$P_{L}=\left\{\begin{array}{c} \frac{2f}{2r}\;\left(For\;spherical\;phase\;with\;a\;radius\;r\right)\\ \frac{3f}{a}\;\left(For\;cubic\;phase\;with\;an\;edge\;length\;a\right)\\ \frac{l+r}{\mathrm{lr}}f\;\left(For\;cylindrical\;phase\;with\;a\;radius\;r\;and\;a\;length\;l\right) \end{array}\right.\;$$

where *f* is the volume fraction of the β‐phase. The shape of the β‐phase has a profound effect on the Al concentration required for the exclusive formation of Al_2_O_3_. This effect is reflected in the influence of shape on *P_L_*. Therefore, *Eff*. is introduced to define the effectiveness of different β‐phase shapes on *PL*. *Eff*. is expressed as: [38]

(10) $$\mathrm{Eff}.=\left\{\begin{array}{c} 3.22\;sphere\\ 4.0\;cube\\ 1.84\sqrt[{3}]{\frac{l}{r}}\left[\frac{r}{l}+\frac{3}{2}\right]\;cylinder \end{array}\right.\;$$

Figure S9 plots the effectiveness (*Eff*.) as a function of aspect ratio (*l/r*) for three different β‐phase shapes. As shown in Figure 1d,e, the shape of β‐phase in the two alloys can be approximated as cylindrical. The measured β‐phase aspect ratios for the two alloys by Image J software are listed in Table S3, the aspect ratio of the β‐phase in MCrAlY and MPEA are 2.36 and 32.05, respectively, and the corresponding *Eff*. of MCrAlY and MPEA are calculated to be 4.71 and 8.95, respectively. The high *Eff*. of MPEA means a reduced minimum Al concentration required for exclusive Al_2_O_3_ formation.

The precipitation of α‐Cr within the β‐phase of the MPEA alloy constitutes a non‐negligible factor in facilitating the establishment of a continuous α‐Al_2_O_3_ scale during the initial oxidation stage. It is well established that both α‐Al_2_O_3_ and Cr_2_O_3_ have the hexagonal close‐packed (hcp) crystal structure [34]. During the initial oxidation stage, nano‐sized α‐Cr particles are preferentially oxidized to form Cr_2_O_3_. Owing to its identical crystal structure to α‐Al_2_O_3_, these Cr_2_O_3_ nanoparticles serve as effective nucleation sites that accelerate the formation of a continuous α‐Al_2_O_3_ scale. This continuous scale subsequently establishes a diffusion barrier that significantly reduces oxygen partial pressure at the oxidation front while suppressing further diffusion of oxygen and metal cations. Consequently, the oxidation process transiting into a stable growth phase is dominated by α‐Al_2_O_3_ scale growth [35].

### 3.2 Mechanism of Retarded Al_2_O_3_ Scale Growth in the Advanced Oxidation Stages

After continuous Al_2_O_3_ scale formation on an alloy surface, the growth of Al_2_O_3_ is achieved by O diffusion through the Al_2_O_3_‐scale and subsequent reaction with Al at the Al_2_O_3_‐scale/substrate interface [34]. With the consumption of Al, an ADZ will form below the interface (Figure S10). To sustain Al_2_O_3_ growth, Al from the underlying substrate must diffuse through this ADZ to replenish the interface. Consequently, the Al diffusion rate within the ADZ directly affect the growth rate of Al_2_O_3_ [43]. The self‐diffusion of metal atoms in FCC phases are governed by the vacancy diffusion mechanism [71]. It has been reported that increased lattice distortion impedes atomic diffusion through elevation of vacancy formation energies and migration barriers [48, 49]. In this study, we designed an MPEA with enhanced configurational entropy and severe lattice distortion relative to MCrAlY. The retarded Al_2_O_3_ growth kinetics in MPEA are directly attributed to this pronounced lattice distortion. To verify the design validity, we quantified lattice distortion in the ADZ and performed first‐principles calculations of vacancy formation energies and Al migration barriers in both alloys.

**FIGURE 6:**
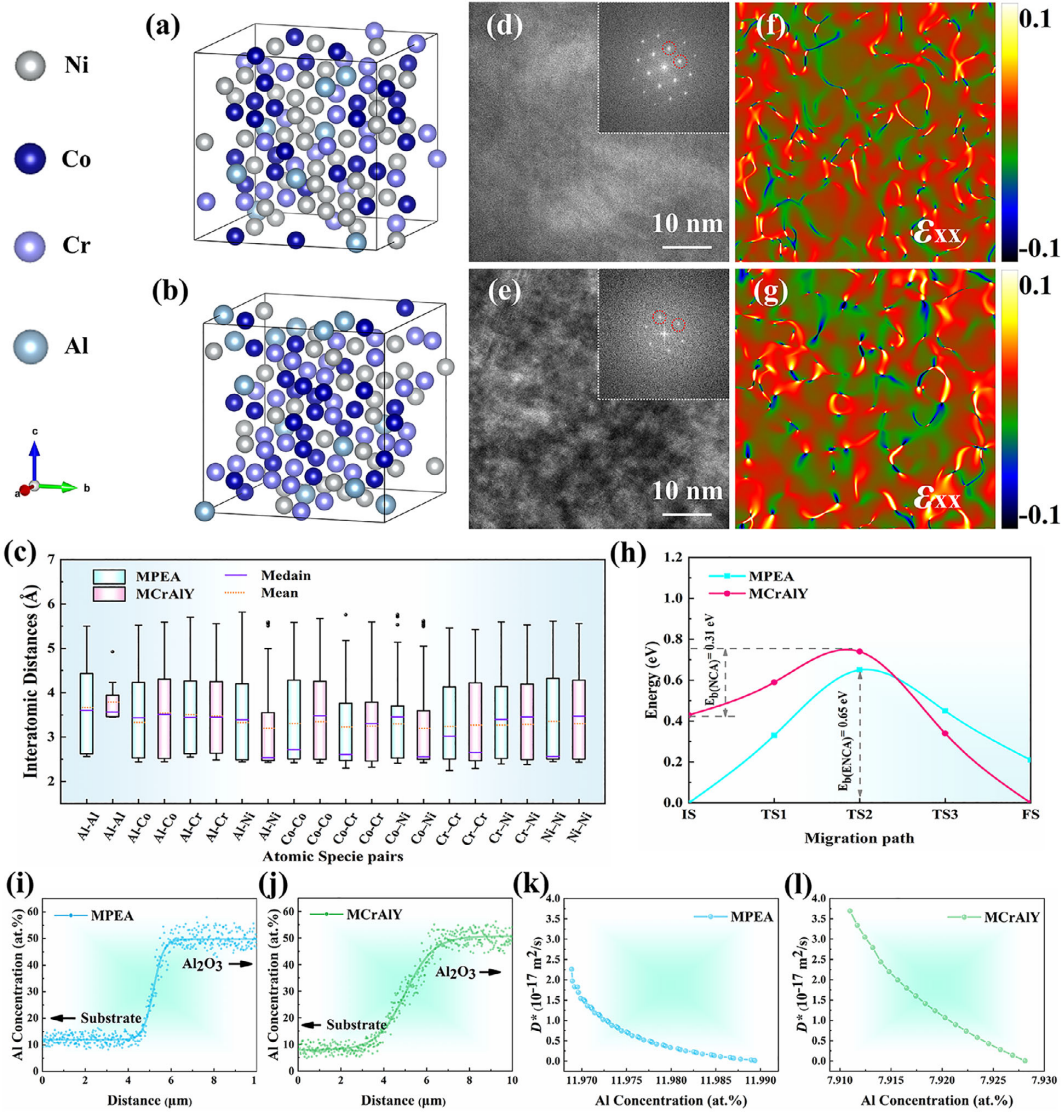
First‐Principles analysis of lattice distortion and Al diffusion rates in ADZ. (a, b): Relaxed supercell structures of simulated ADZ in MCrAlY (a) and MPEA (b) exhibit total system energies of ‐731.94 and ‐748.84 eV, respectively; (c): The box plot shows the distribution of interatomic distances of 1NN bonds in the supercell structures. The box indicates the range from the first and third quantiles, the whisker extends to 1.5 times the interquartile ranges, and the dots represent outliers beyond the whiskers; d, (e): HRTEM images of ADZ: (d) MCrAlY and (e) MPEA, the inset displays the corresponding FFT pattern (The red circles denote the two g‐vectors used for GPA); (f, g): Atomic scale strain distribution maps via GPA of HRTEM images: (f) MCrAlY and (g) MPEA; (h): Energy barrier diagram for Al diffusion in ADZ calculated via CI‐NEB method reveal significantly higher diffusion barriers in MPEA (0.65 eV) versus MCrAlY (0.31 eV); (i, j): Experimentally obtained Al concentration profiles from the ADZ to the Al_2_O_3_ scale for the MPEA and MCrAlY alloy, respectively; (k, l): The concentration‐dependent diffusion coefficients, D(c*), for the MPEA and MCrAlY, derived from the concentration profiles in (i) and (j), respectively.

The chemical composition and configurational entropy of the ADZ in the two alloy is listed in Table S4. Based on the chemical compositions of the ADZ in the two alloys, 3 × 3 × 3 FCC supercells modeling their ADZ were constructed. Following lattice relaxation, the energy‐minimized supercell structures depicting ADZ in MPEA and MCrAlY are presented in Figure 6a,b, respectively. Distribution statistics of first nearest‐neighbor (1NN) bond lengths for distinct atomic‐species pairs are presented in Figure 6c. Majority of atom‐species pairs in the ADZ supercell structure of MPEA exhibit significantly broader 1NN bond length distributions compared to their MCrAlY counterpart, manifesting enhanced lattice distortion within MPEA's ADZ. Lattice distortion within the ADZ of both alloys was subsequently quantitatively evaluated using the method proposed by Lee et al. The lattice distortion ${\bar{u}}^{D}$ can be expressed as: [72]

(11) $${\bar{u}}^{D}=\sqrt{\sum_{i}^{n}{\left(d_{i}^{eff}-\bar{d}\right)}^{2}/n}\;$$

where *deff i* is the effective interatomic distance of the i th element, and $\bar{d}$ is average interatomic distance of n elements, and n is the number of constituent elements. Parameters *deff i* and $\bar{d}$ are computationally extracted by statistically analyzing 1NN bond lengths across all atomic‐species pairs in the supercell. The ${\bar{u}}^{D}$ in the ADZ is calculated as 0.0862 Å for the MPEA and 0.0591 Å for the MCrAlY. The detailed descriptions of the quantitative estimation of the ${\bar{u}}^{D}$ are explained in supplementary Note S2 and Figure S11.

Notably, both bond length distribution statistics and quantitative lattice distortion estimation demonstrate greater lattice distortion in the ADZ of the MPEA compared to MCrAlY. To experimentally validate the lattice distortion simulations, GPA was employed to map atomic‐scale in‐plane normal strain (*ε_xx_*) distributions within the ADZs of two alloys, as shown in Figure 6d–g. ImageJ quantification of high‐strain regions reveals significantly larger strained areas in MPEA's ADZ (21.3%) versus MCrAlY (14.8%), representing enhanced lattice distortion in MPEA, which is consistent with the simulation results.

Aberration‐corrected STEM was employed to directly examine the ADZ in both alloys (Figure S12), providing further evidence for the difference in their lattice distortion levels. Atomic‐resolution HAADF‐STEM imaging and EDX mapping along the [110] zone axis (Figure S12a,b) reveal a random distribution of Ni, Co, Cr, and Al in the FCC lattice. Critically, the MPEA exhibits a smaller (200) interplanar spacing (1.864 Å) than the MCrAlY alloy (1.875 Å). Given the predominantly compressive nature of the lattice strain identified by GPA, this reduced d‐spacing provides a direct metric for the greater lattice distortion in the MPEA [73, 74]. This finding is visually confirmed by IFFT analysis (Figure S12a_2_ and S12b_2_), where the MPEA contains a notably higher density of local distortion regions.

Subsequently, we analyzed Al diffusion kinetics in the ADZ of the two alloys through computational determination of vacancy formation energies and Al atom migration energy barriers. The vacancy formation energy (*E_f_*) was determined by comparing the total energy of a relaxed perfect FCC supercell with that of a relaxed supercell containing a Ni vacancy at the nearest neighbor site to an Al atom. *E_f_* is expressed as: [49, 75]:

(12) $$E_{f}=E_{i}+E_{0}+\mu_{i}$$

where *E_i_* and *E_0_* are the total energy of the supercell with and without the vacancy, respectively, *µ_i_* is the chemical potential of species i, with *µN_i_* = ‐5.48 eV in this study. The detailed computational methodology is provided in the Supplementary Note 3. *E_0_* was obtained from the relaxed defect‐free supercell structures (Figure 6a,b) used for lattice distortion evaluation, with values of ‐748.84 eV for MPEA and ‐731.94 eV for MCrAlY. Supercells containing single vacancies were created through targeted removal of Ni atoms at Al 1NN sites in pristine FCC structures (Figure S13). Post‐relaxation *E_i_* of these ADZ structural models measured ‐741.48 eV (MPEA) and ‐724.40 eV (MCrAlY). As shown in Figure S13a,b, our computational model features an Al atom positioned at the origin [0 0 0] with a Ni vacancy created at its 1NN site, a configuration designed for visualization clarity. Consequently, the vacancy formation energies in the ADZ of MPEA and MCrAlY were calculated as 1.98 eV and 1.84 eV, respectively.

The migration barriers of Al atom in ADZ of MPEA and MCrAlY was calculated using the climbing image nudged elastic band (CI‐NEB) method. The configurations with one Ni vacancy served as the initial stable state for the migration of Al atom (Figure S13a,b). The final stable state was configured with an Al atom migrated into the 1NN vacancy site (Figure S13c,d). Three intermediate configurations were linearly interpolated between the two stable states, yielding the Al migration energy barrier as shown in Figure 6h. It is evident that the migration energy barrier for Al diffusion in ADZ of MPEA (0.65 eV) is higher than in ADZ of MCrAlY (0.31 eV).

To accurately determine the aluminum diffusion coefficient within the ADZ of the two alloys, the concentration‐dependent diffusion coefficient D(c*) was then determined via Boltzmann‐Matano analysis [76, 77]. A detailed description of the Boltzmann‐Matano method is provided in Note 4 of Supporting Information. Using the Al concentration profiles in the ADZ of the MPEA and MCrAlY alloy after 10 h of oxidation (Figure 6i,j, respectively), we determined the concentration‐dependent Al diffusion coefficients within the ADZs via the Boltzmann‐Matano method. The results are shown in Figures 6k,l. The results show that, for the MPEA, the diffusion coefficient of Al in the depleted zone ranges from 1.3×10^−19^ m^2^/s to 2.3×10^−17^ m^2^/s, with a mean value of 7.7×10^−18^ m^2^/s. In comparison, the commercial NiCoCrAlY alloy exhibits aluminum diffusion coefficients ranging from 6.3×10^−20^ m^2^/s to 3.6×10^−17^ m^2^/s in its ADZ. The average diffusion coefficient in the commercial alloy is calculated to be 1.6×10^−17^ m^2^/s, which is significantly higher than that of the MPEA.

The above analysis confirms the presence of greater lattice distortion in the ADZ of the MPEA, along with its higher vacancy formation energy, migration energy, and consequently, suppressed diffusivity relative to the MCrAlY alloy. These findings collectively confirm the effectiveness of our alloy design strategy.

## 4 Conclusion

In this study, we propose a novel dual‐pronged strategy to enhance alloy oxidation resistance: 1) promoting selective oxidation of aluminum (Al) during the initial oxidation stage, and 2) suppressing Al diffusion toward the oxidation front in the advanced oxidation stages. Guided by this approach, we successfully designed a MPEA which exhibits outstanding oxidation resistance at 1200°C, manifested through significantly retarded oxide scale growth kinetics and exceptional scale spallation resistance. Analyses of the initial oxidation behavior reveal that the lamellar microstructure of MPEA lowers the critical Al concentration required for exclusive Al_2_O_3_ formation. This enables rapid establishment of a protective, exclusive Al_2_O_3_ scale during the initial oxidation stage. First‐principles calculations of Al diffusion kinetics across the ADZ demonstrate that entropy‐enhanced lattice distortion within ADZ in MPEA distinctly elevates vacancy formation energy and Al migration barrier, suppressing scale growth. In summary, the exceptional oxidation resistance of MPEA at 1200°C, combined with its identical base constituting elements to the MCrAlY benchmark, establishes it as a highly promising candidate material for next‐generation thermal barrier coating bond coats. Furthermore, the dual‐mechanism design strategy presented in this work provides a valuable paradigm for developing advanced oxidation‐resistant materials.

## 5 Materials and Methods

Materials and methods can be found in the supporting information.

## Author Contributions

X.Y.Z. conceived the material composition, conducted all experimental work, performed data curation and investigation, and drafted the original manuscript. W.Y.L. provided conceptualization, designed the experiments, assisted in data compilation, and contributed significantly to revising the manuscript through review and editing. X.G.W. performed the TEM characterization and corresponding data analysis. C.M.J. conducted the first‐principal calculations and corresponding analysis. Y.Z.Z. supported the structural analysis of the MPEA. K.Q.Q. contributed to developing the experimental methodology and performing data analysis, and participated in reviewing and editing the manuscript. J.Q.W. oversaw project administration, supervision, resource provision, validation, visualization, and contributed to manuscript review and editing. All the authors contributed to the discussion and revision of the manuscript.

## Conflicts of Interest

The authors declare no conflict of interest.

## Supporting information




**Supporting File**: advs74168‐sup‐0001‐SuppMat.docx.

## Data Availability

The data that support the findings of this study are available from the corresponding author upon reasonable request.
