# Supplementary material for: Outstanding 1200 °C Oxidation Resistance in a Novel Multi‐Principal Element Alloy via Lattice Distortion‐Induced Diffusion Suppression
Source: Adv Sci (Weinh). 2026 Feb 6;13(20):e22526. doi: 10.1002/advs.202522526 (PMC13067856; doi:10.1002/advs.202522526)
Supplement: Supplementary file 1 — Supporting File: advs74168‐sup‐0001‐SuppMat.docx. [file ADVS-13-e22526-s001.docx]

**Supplemental information**

**Outstanding 1200 ℃ Oxidation Resistance in a Novel Multi-Principal Element Alloy via Lattice Distortion-Induced Diffusion Suppression**

Xinyu Zhang ^a, b^, Weiyan Lv ^a, b,^ *, Xinguang Wang ^a^, Chuanmin Jia ^c^, Yizhou Zhou ^a^, Keqiang Qiu ^b,^ *, Jianqiang Wang ^a,^ *

**^a^** *Shenyang National Laboratory for Materials Science, Institute of Metal Research, CAS, Shenyang 110016, P. R. China*

**^b^** *School of Materials Science and Engineering, Shenyang University of Technology, Shenyang 110870, P.R. China*

**^c^** *Wangxuan Institute of Computer Technology, Peking University, Beijing 100871, P.R. China*

* Corresponding author,

E-mail: wylv@imr.ac.cn (Weiyan Lv)

kqqiu@sut.edu.cn (Keqiang Qiu)

jqwang@imr.ac.cn (Jianqiang Wang)

**Supplemental Note 1. Experimental Procedures**

**Alloy Design**

To achieve enhanced configurational entropy and lattice distortion relative to conventional NiCoCrAlY alloys, we designed a NiCoCrAl-type Multi-Principal Element alloy (MPEA) with elevated Co and Cr content, denoted as Ni_50-x_Co_25_Cr_25_Al_x_. The pseudo-binary phase diagram of this system was calculated using the CALPHAD methodology implemented in Thermo-Calc (TC) software,^[1]^ identifying a eutectic point at the Ni_32_Co_25_Cr_25_Al_18_ composition.

**Sample preparation**

The designed Ni_32_Co_25_Cr_25_Al_12_ (at.%) alloy (MPEA) doped with reactive elements (0.1 wt.% Y and 0.1 wt.% Hf) was prepared by arc-melting high-purity (99.99 wt. %) Ni, Co, Cr, Al, Y and Hf metals under a protective argon atmosphere. For comparative experiments, a commercial NiCoCrAl alloy (MCrAlY) with equivalent Y-Hf additions was prepared by arc-melting, with the nominal composition Ni_48_Co_23_Cr_17_Al_12_ (wt.%). Each ingot has a weight of about 150 g. To increase the homogeneity of the prepared alloy, the melting process was conducted five times.

**Isothermal oxidation experiment**

Isothermal oxidation specimens were sectioned by wire-electrode cutting into 5 mm × 5 mm × 5 mm cubes. Prior to oxidation testing, all surfaces were ground with SiC abrasive paper up to 2000-grit, followed by ultrasonic cleaning in acetone. After thorough drying, each specimen was weighed using an analytical balance with 0.01 mg precision, and the initial mass was recorded. Isothermal oxidation experiments were conducted at 1200 ℃ in ambient atmosphere using a muffle furnace for 500 h. Crucibles containing specimens were positioned in a muffle furnace at 1200 ℃. At predetermined time intervals, crucibles were extracted and forcibly air-cooled to room temperature using an electric fan, then specimens were removed for mass measurement. Each specimen was weighed at least three times to ensure measurement reproducibility.

**Cyclic oxidation experiment**

Specimens were wire-cut into 5 mm × 5 mm × 5 mm cuboids. Prior to cyclic oxidation testing, all surfaces were ground to 2000-grit using SiC abrasive paper, ultrasonically cleaned in acetone (15 min), and thoroughly dried. Crucibles containing specimens were placed in a muffle furnace at 1200 ℃ for 12 h, followed by forced-air cooling to room temperature within 10 min using an electric fan – defining one thermal cycle. This cycle was repeated for cumulative 504 h exposure (42 cycles). At 12 h intervals, specimens were photographically documented under calibrated stereomicroscopy. Oxide scale spallation area fractions were quantified via ImageJ software.^[2]^ Functional failure was defined as ≥15% spalled surface area.

**Tensile testing**

To evaluate the tensile properties, monotonic tension tests were performed at room temperature under quasi-static conditions (strain rate: 1×10^-3^ s^-1^) using an Instron 5848 machine. The tests utilized dog-bone-shaped specimens with gauge dimensions of 20×4×1.5 mm^3^.

**Samples characterization**

Phase structures of the alloys were characterized by X-ray Diffraction (XRD, Carl Zeiss SUPRA 55 SAPPHIRE, Germany) using Cu Kα radiation (λ=1.5406Å), with a scanning rate of 5°/min over a 2θ range of 10-90°. Microstructure and elemental distribution of specimens and oxide scales were characterized using Field-emission Scanning Electron Microscopy (FESEM, Carl Zeiss SUPRA 55 SAPPHIRE, Germany) in Backscattered Electron (BSE) mode equipped with Energy-Dispersive X-ray Spectroscopy (EDS). The extent of oxide scale spallation on cyclic oxidation samples was quantitatively characterized using Stereomicroscopy (Zeiss Stemi508, Germany) coupled with ImageJ software analysis. Microstructure and elemental distributions of alloys and oxide scales were identified more detailed using Analytical Scanning Transmission Electron Microscopy (STEM, Japan Electron Optics Laboratory JEM-F200, Japan) equipped with a Super-X Energy-Dispersive X-ray Spectroscopy (EDS) system. The lattice distortion and elemental distribution in the Al-depletion zones were examined at atomic resolution using aberration-corrected scanning transmission electron microscopy (STEM, ThermoScientific Spectra200 USA). The TEM specimen was prepared by the SEM microscope equipped with a Ga focused-ion-beam (FIB, 30 kV, Thermo Scientific Scios 2) and an Omniprobe manipulator. The pieces were initially pre-cut from the bulk samples by using a current of 7 nA, and then ion beam current from 0.5, 0.3, 0.1 nA to 10 pA were used in sequence to further mill the piece into electron-transparent slices with thickness of 70 nm. The residual stress within the oxide scale was measured via photoluminescence piezospectroscopy (PLPS) method on a Confocal Raman microprobe (LabRAM Odyssey, Horiba, Japan) equipped with an Olympus Microscope and 532 nm Nd:YAG laser source.

**CALPHAD method**

The pseudo-binary phase diagram of Ni_50-x_Co_25_Cr_25_Al_x_ (at.%) system and phase fractions of Ni_32_Co_25_Cr_25_Al_18_ (at.%)was calculated by the CALPHAD method. The TCNI9 thermodynamic database by Thermo-Calc. To elucidate phase composition differences between the designed MPEA and commercial NiCoCrAl alloys, pseudo-binary phase diagrams of Ni_60-x_Co_23_Cr_17_Al_12_ (wt.%) system and phase fraction of Ni_48_Co_23_Cr_17_Al_12_ (wt.%) were calculated shown in Figure S1. Additionally, aluminum diffusion rates in the γ-phase were simulated using Thermo-Calc (TC) software shown in Figure S8.

**Theoretical Calculation**

First-principles calculations were carried out using the projector augmented-waves (PAW) method as implemented in the Vienna Ab initio Simulation Package (VASP).^[3]^ The generalized gradient approximation (GGA) parameterized by Perdew-Burke-Ernzerhof (PBE) was used for the exchange-correlation functional. A cutoff energy of 400 eV was used to diminish the expansion of plane-wave basis sets. The Monkhorst-Pack method was used to generate the k-point meshes with the density of ~0.02Å^−1^. All structures were fully relaxed with respect to atomic positions, box volumes and box shapes through minimizing the total energies until the Hellmann-Feynman forces on all atoms were below 0.01 eV/Å. Transition states were searched using the climbing image nudged elastic band (CI-NEB) method as implemented in the VTST package.

**Supplemental Note 2. Details of quantitative evaluation of lattice distortion**

In an ideal FCC unit cell composed of identical atoms, the effective interatomic distance *deff i* of the constituent elements equals its average interatomic distance$\bar{d}$. This ideal atomic arrangement implies the absence of lattice distortion, resulting in a value of $\bar{u}^{D}$ = 0 in Equation (11) of the main text. However, in FCC multi-principal element alloys, atomic size mismatch and varying interatomic interactions between constituent elements generate divergent atomic spacings, thereby inducing lattice distortion as illustrated in Figure S11. Within such distorted crystal structures, the deviation between *deff i* and $\bar{d}$ quantifies the average lattice distortion $\bar{u}^{D}$ in the FCC alloy system. The average lattice distortion $\bar{u}^{D}$ can be express as:^[4-6]^

$$\bar{u}^{D}=\sqrt{{\sum_{i}^{n} {(d_{i}^{eff}-\bar{d})}^{2}}/n}$$

where *deff i* is the effective interatomic distance of the ith element, and $\bar{d}$ is average interatomic distance of n elements, and n is the number of constituent elements. Parameters *deff i* and $\bar{d}$ are calculated by statistically analyzing 1NN bond lengths across all heteroatomic-species pairs in the supercell. The detailed calculation procedure is depicted in Figure S11, and the corresponding results are listed in Table S5.

**Supplemental Note 3. Approaches to calculating chemical potential *μ_i_***

The chemical potential *μ* is defined as the energy change associated with adding or removing one atom from a system. For pure metals, this quantity is typically calculated as the energy per atom of the respective element. Literature suggests that the variation in *μ* between pure metals and alloys is negligible.^[7-9]^ Consequently, to minimize computational demands, the distinction in chemical potential between pure metals and alloys was neglected in this work. An FCC Ni unit cell was constructed and fully relaxed, yielding a total energy of -0.21939217 × 10^2^ eV for the system. The chemical potential of Ni (*μ_Ni_*) was then determined as -5.48 eV per atom by dividing the total energy by the number of atoms in the unit cell (N = 4).

**Supplemental Note 4. Detailed description of the Boltzmann-Matano method**

To accurately determine the aluminum diffusion coefficient within the ADZ of the two alloys, the region adjacent to the oxide scale after 10 hours of oxidation was treated as a diffusion couple. The Al concentration profile, spanning from the ADZ to the Al_2_O_3_ scale, was acquired by EDS and is presented in Figure 6i and 6j (Main Text). This profile was subsequently fitted using a Boltzmann function in Origin software to generate a smoothed concentration curve. The concentration-dependent diffusion coefficient *D(c*)* was then determined via Boltzmann-Matano analysis:^[10,11]^

$$D\left( c^{*} \right)=-\frac{1}{2}\frac{\int_{c^{*}}^{c^{R}} \left( x-x_{0} \right)dc}{\left( {dc}/{dx} \right)_{c=c^{*}}}$$

where *D(c*)* represents the diffusion coefficient (m^2^/s) at concentration *c**, *t* denotes the oxidation time (*t* = 36000 s in this study), *x* is the spatial coordinate (m), *x_0_* indicates the Matano interface position (m), *c_R_* corresponds to the atomic percentage of Al in Al_2_O_3_, and (*dc/dx*) describes the concentration gradient evaluated at concentration *c**. The concentration-dependent diffusion coefficients within the ADZ of the MPEA and the commercial NiCoCrAlY alloy were determined from the concentration profiles in Figures 6i and 6j (Main Text) using the aforementioned equation, with the results presented in Figures 6k and 6l of the Main Text, respectively.


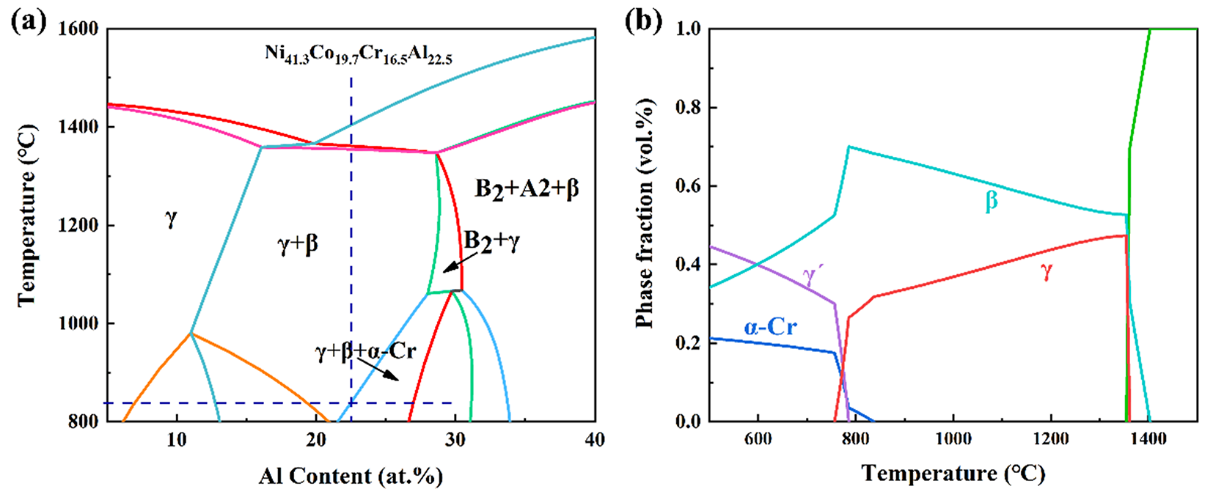


**Figure S1.** a): Pseudo-binary phase diagram of Ni_63.8-x_Co_19.7_Cr_16.5_Al_x_ (at.%)system calculated using the CALPHAD method; b): Phase fraction of Ni_41.3_Co_19.7_Cr_16.5_Al_22.5_ (at.%) calculated using the CALPHAD method.


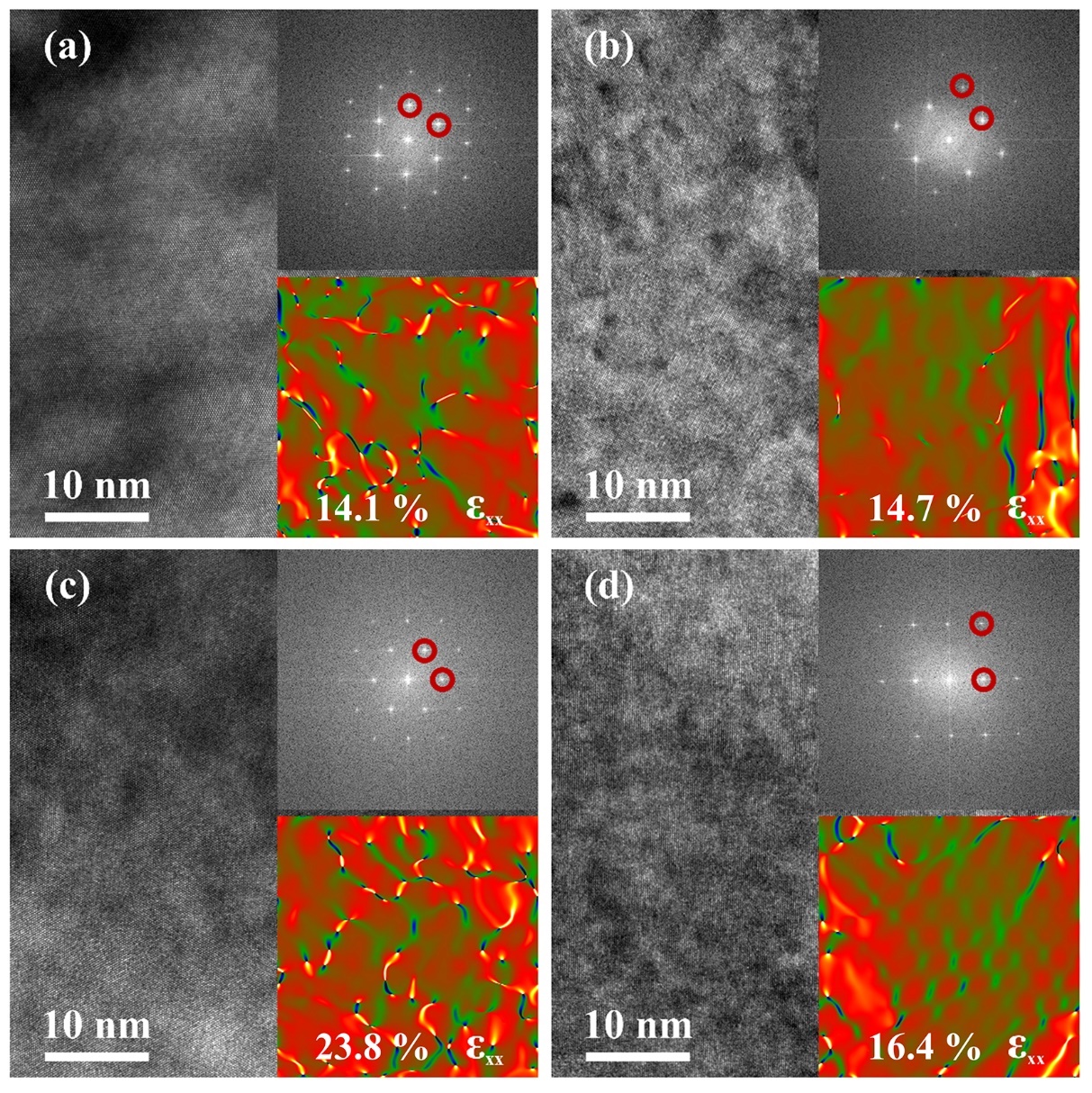


**Figure S2.** a, b):HRTEM image of β-phase (a) and γ-phase (b) in MCrAlY with corresponding FFT pattern (upper right inset) and atomic-scale strain distribution map (lower right inset) obtained via geometric phase analysis of HRTEM data; c, d): HRTEM image of β-phase (a) and γ-phase (b) in MCrAlY with corresponding FFT pattern (upper right inset) and atomic-scale strain distribution map (lower right inset) obtained via geometric phase analysis of HRTEM data (The red circles denote the two g-vectors used for GPA).


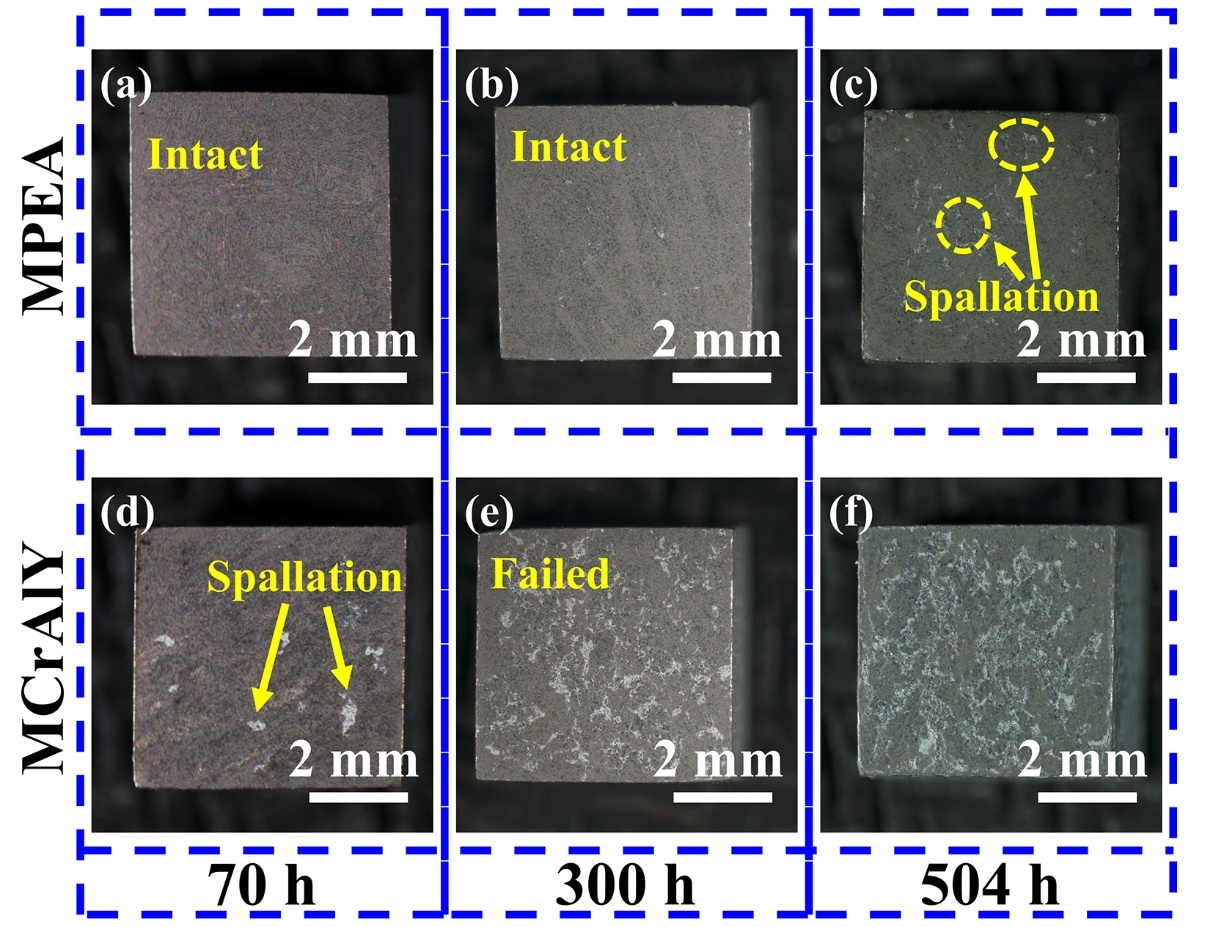


**Figure S3.** Macroscopic progression of TGO spallation in (a-c) MPEA and (d-f) MCrAlY following cyclic oxidation at 70 h, 300 h, and 504 h intervals. Images acquired by stereomicroscopy.





**Figure S4.** Room-temperature tensile stress-strain curves of MPEA and MCrAlY.


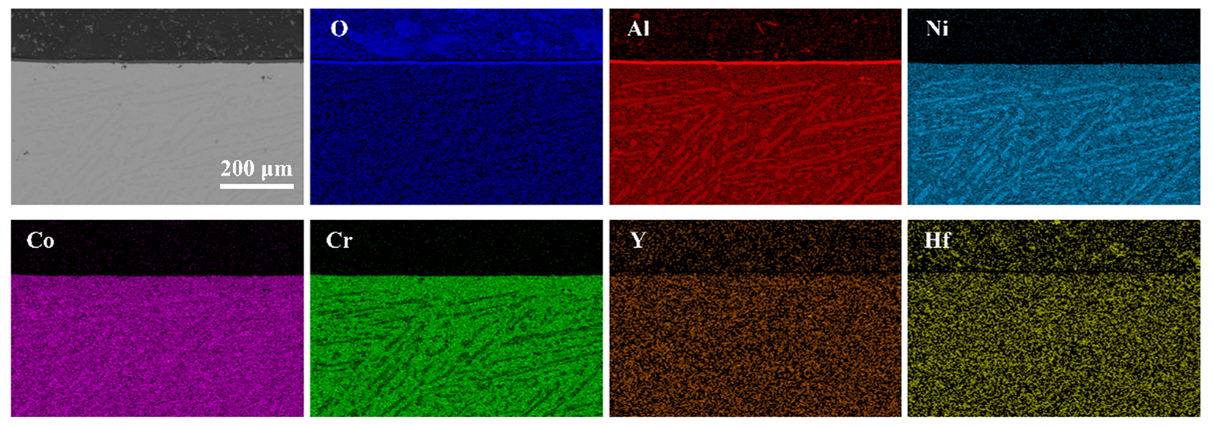


**Figure S5.** Cross-sectional SEM image of MPEA oxide scale and corresponding elemental mappings, revealing Al- and O-rich oxide scale identified as Al_2_O_3_.


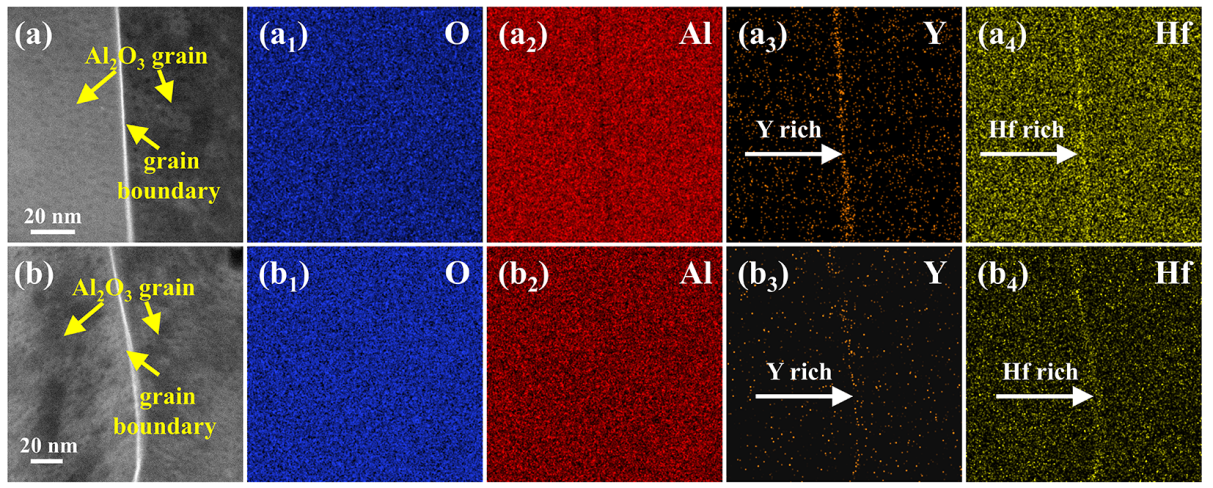


**Figure S6.** STEM images showing Al_2_O_3_ grains and grain boundaries along with their corresponding elemental distribution maps for the two alloys: (a) MPEA and (b) MCrAlY.


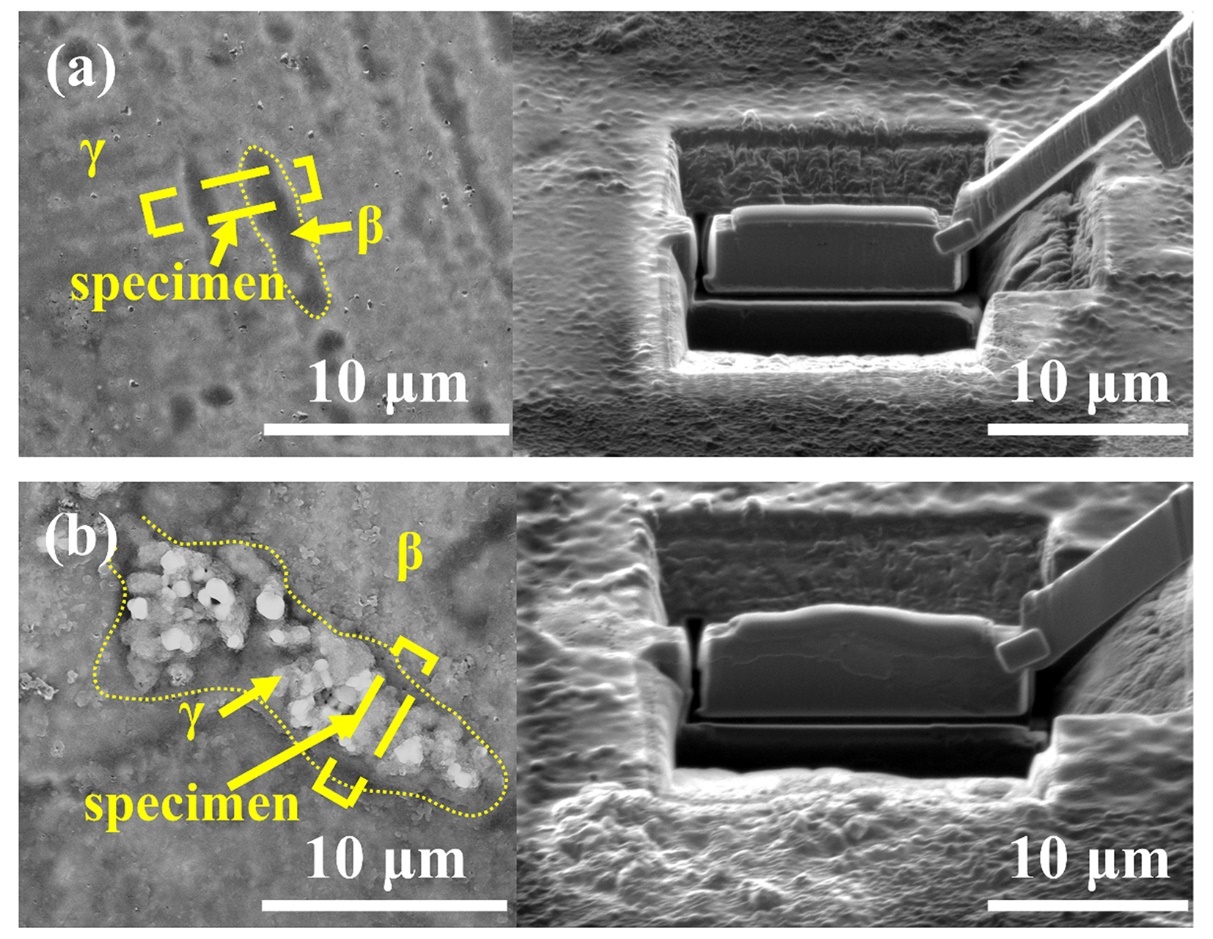


**Figure S7.** Sampling positions for TEM characterization at initial oxidation stage: (a) MPEA, (b) MCrAlY. (The selected sampling positions incorporated oxide scale formation on both β-phase and γ-phase surfaces.)

**
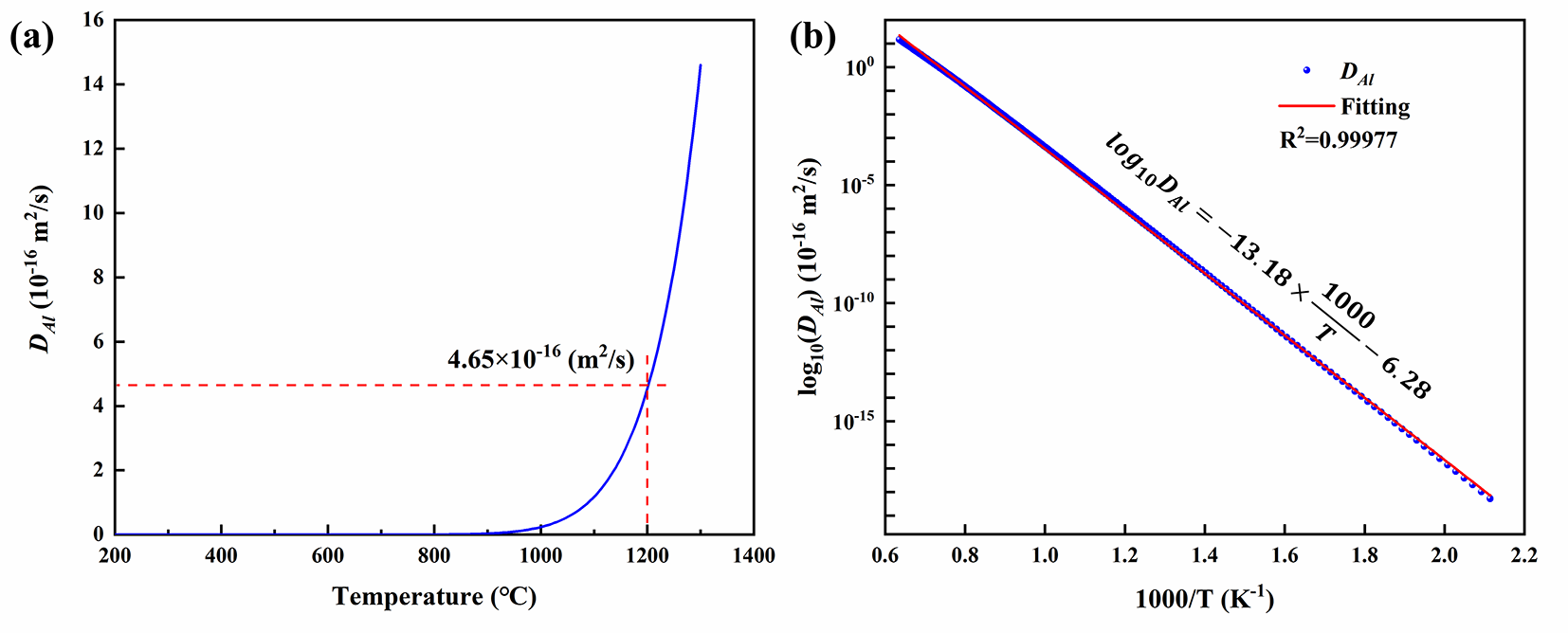
**

**Figure S8.** a):Temperature-dependent diffusion rate curve of Al atoms in γ-phase calculated using Thermo-Calc software; b): Arrhenius plot of the Al diffusion coefficient *(D_Al_)* in the γ phase as a function of temperature (*T*).


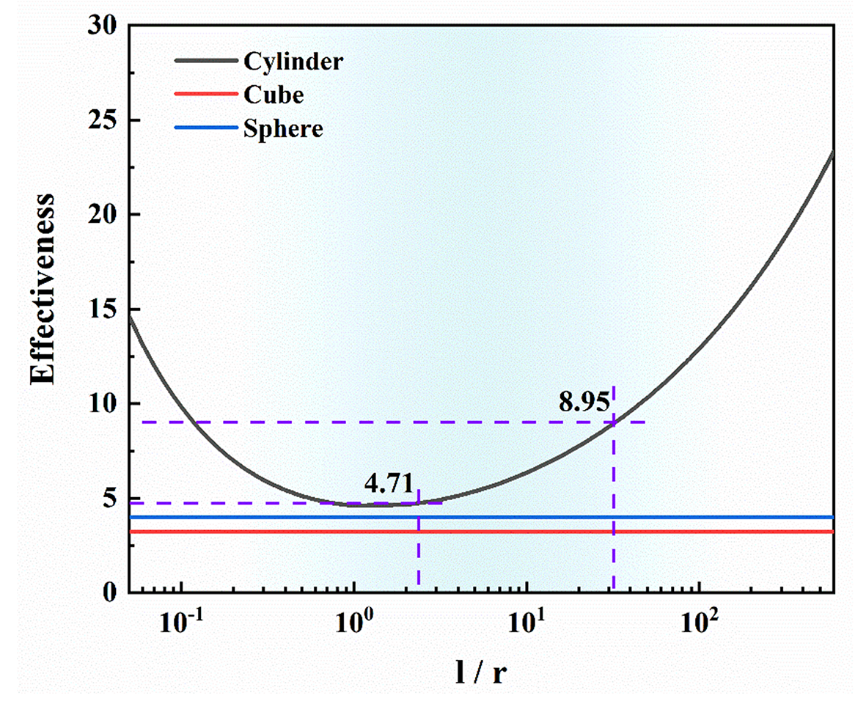


**Figure S9.** Effectiveness (*Eff.*) of Al₂O₃ exclusive formation vs. aspect ratio (*l/r*) in β-phase of varying geometries (spherical, cubic, and cylindrical).


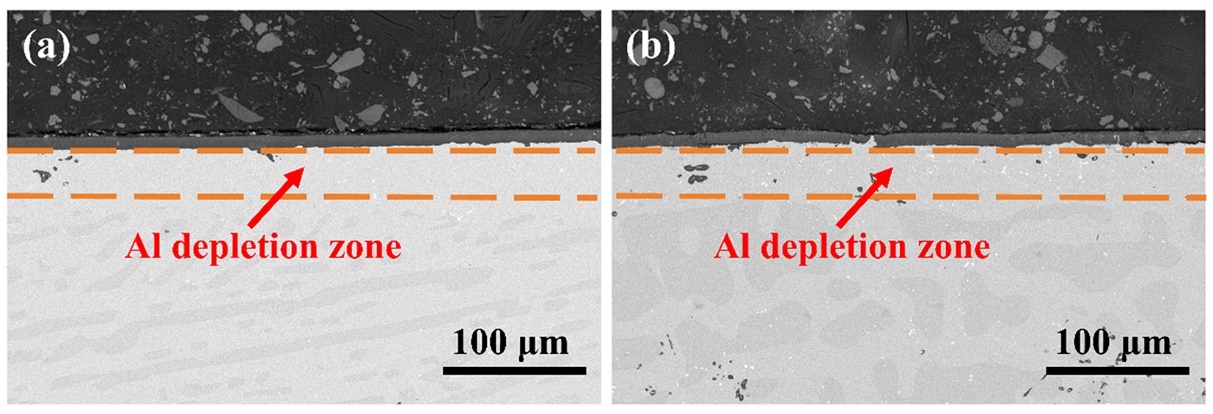


**Figure S10.** a): Cross-sectional SEM image of MPEA oxide scale; b): Cross-sectional SEM image of MCrAlY oxide scale. Al depletion zones of comparable thickness formed beneath the oxide scales in the two alloys.


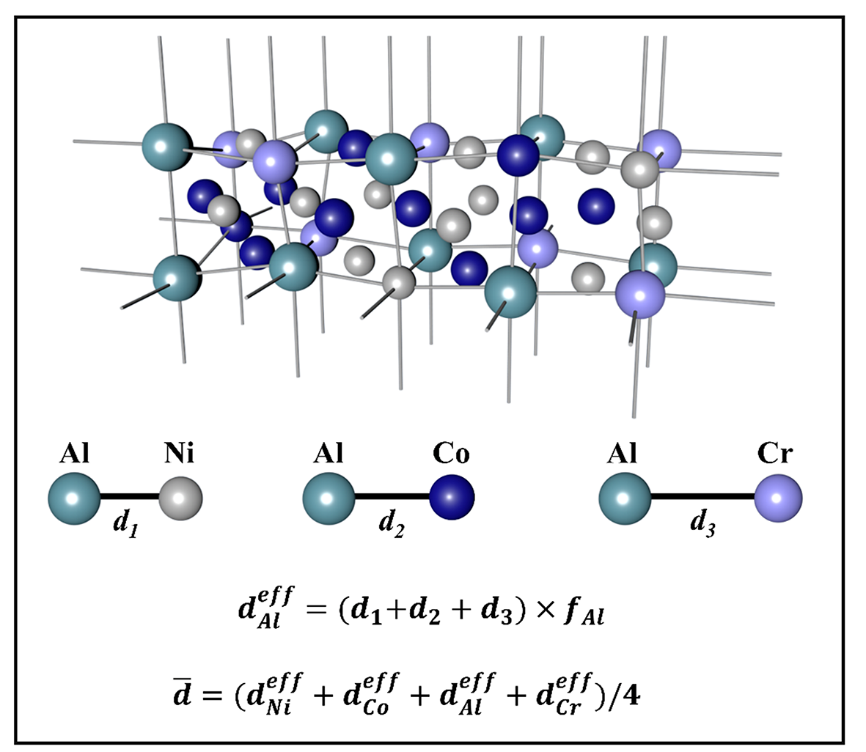


**Figure S11.** Schematic illustration for calculating the effective interatomic distance (*deff i*) of constituent elements and average interatomic distance ($\bar{d}$).


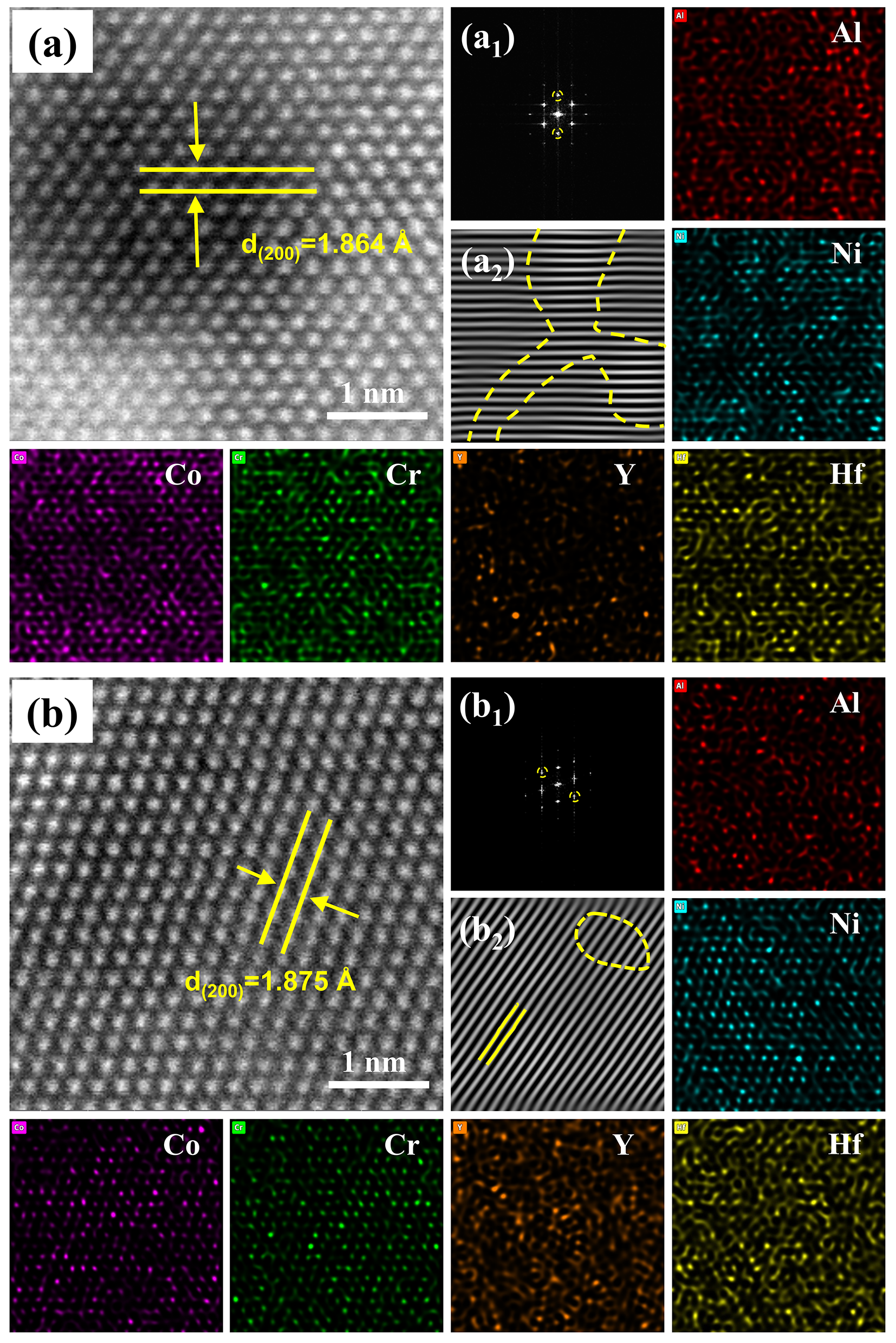


**Figure S12.** Atomic-scale analysis of lattice distortion and elemental distribution in the Al-depletion zones of the two alloys; a): Atomic-resolution HAADF-STEM image of MPEA; a_1_): Corresponding FFT pattern, with the yellow circle highlighting the (200) diffraction spot used for analysis; a_2_): IFFT image, where yellow regions indicate areas of continuous lattice distortion; b): Atomic-resolution HAADF-STEM image of the commercial MCrAlY; b_1_): Corresponding FFT pattern, with the (200) spot selected (yellow circle); b_2_): IFFT image revealing localized lattice distortion (yellow regions).


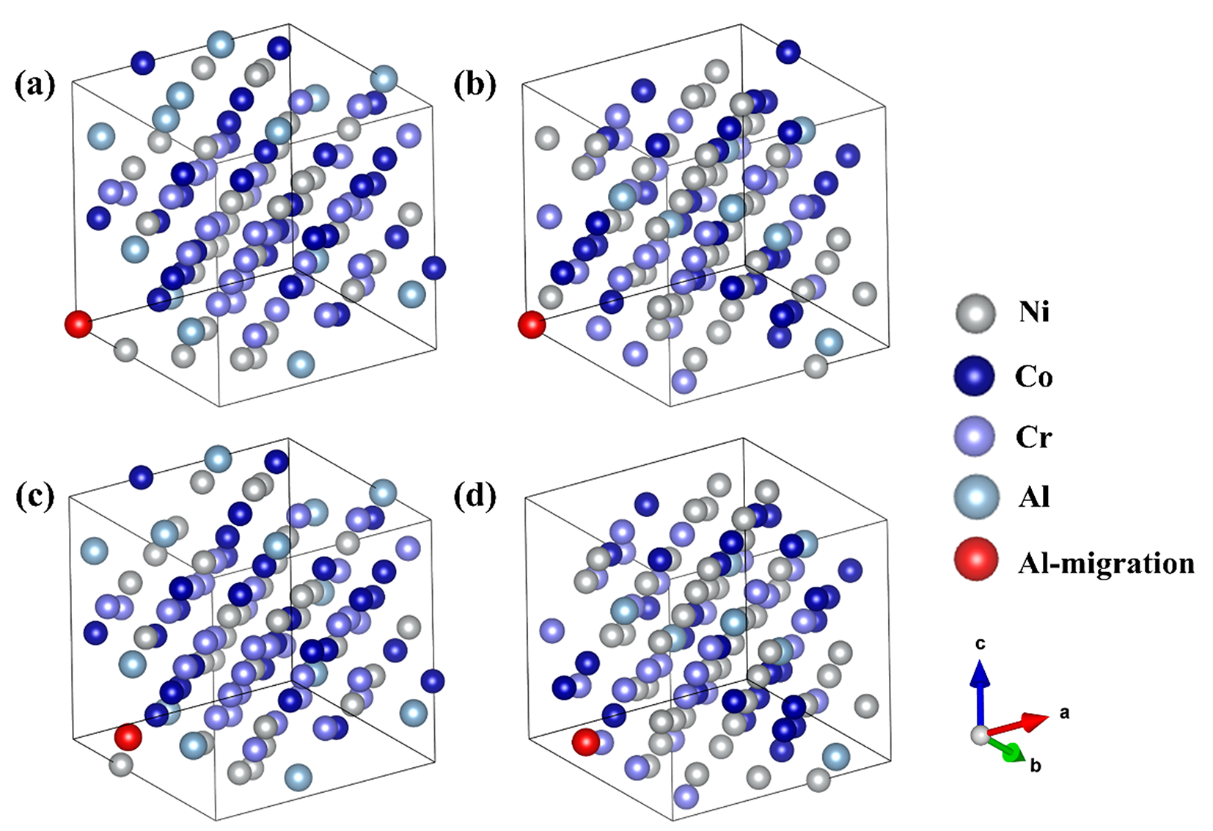


**Figure S13.** a, b): Supercell structures of Al-depletion zones with a Ni vacancy at the [0.5, 0.5, 0] position: (a) MPEA, (b) MCrAlY. (These supercells serve as initial configurations for Al migration, with migrating Al atoms marked in red); c, d): Terminal configurations of Al-depletion supercells after Al atom migration to the [0.5, 0.5, 0] vacancy site: (c) MPEA, (d) MCrAlY. (These supercells represent the final state of Al migration).

**Table S1.** Detailed data of Figure 2c in the main text, including oxide rate constant (*k_p_*) of this work and other Al_2_O_3_-forming alloys in the literature available.

| Number | Materials | Oxide rate constant  kp (10-12 g·cm4·s-1) | Ref. |
| --- | --- | --- | --- |
| 1 | MPEA | 1.28 | This work |
| 2 | NiCoCrAlPt | 1.3 | 12 |
| 3 | NiCoCrAlYHf | 2.9 | 13 |
| 4 | CoNiCrAlY | 16.5 | 14 |
| 5 | Al_18_Co_30_Cr_10_Fe_10_Ni_32_ | 1.33 | 13 |
| 6 | Al_1.1_CoCr_0.8_FeNi | 1.4 | 15 |
| 7 | Al_13.0_Cr_21.7_Fe_21.7_Ni_21.8_Co_21.7_ | 6 | 16 |
| 8 | Fe-13.04Cr-5.61Al-0.032Y | 2.96 | 17 |
| 9 | Kanthal APM alloy | 3 | 18 |
| 10 | FeCrAl-0.02 at.%Y+Hf | 3.5 | 19 |
| 11 | Kanthal AF alloy | 4 | 20 |
| 12 | FeCrAl -0.038 at.%Y+Hf | 5.6 | 19 |
| 13 | FeCrAl-0.08 at.%Y | 7.5 | 19 |
| 14 | Kanthal AF alloy | 8.61 | 21 |
| 15 | Fe-13Cr-6Al-2Mo-0.5Nb+0.01(wt.%)Y | 9.83 | 22 |
| 16 | FeCrAl | 11 | 18 |
| 17 | Fe-13Cr-6Al-2Mo-0.5Nb+0.1(wt.%)Y | 11.69 | 22 |
| 18 | Fe-13Cr-6Al-2Mo-0.5Nb+0.2(wt.%)Y | 13.33 | 22 |
| 19 | Fe-13.80Cr-6.2Al-0.1Y-0.1Mo-0.2Ti | 15.21 | 17 |
| 20 | Fe-13Cr-6Al-2Mo-0.5Nb | 16.5 | 22 |
| 21 | Fe-22Cr-5Al-3Mo-2Nb | 25.15 | 23 |
| 22 | Fe-22Cr-5Al-3Mo-1Nb | 32.5 | 23 |
| 23 | NiAl-1Cr+Hf | 1.3 | 24 |
| 24 | NiAlZr | 1.53 | 25 |
| 25 | NiAlHf-1Si | 1.64 | 26 |
| 26 | NiAl-1Ta+Hf | 1.7 | 24 |
| 27 | NiAlHf-3Si | 2.09 | 26 |
| 28 | NiAl-1Ti+Hf | 2.2 | 24 |
| 29 | Ni-Al49.9-Hf0.05-Zr0.05 | 2.26 | 27 |
| 30 | NiAlZr0.04 | 2.4 | 28 |
| 31 | Ni-Al49.9-Hf0.1 | 2.94 | 27 |
| 32 | NiAl-0.05Hf-0.05Dy | 3.17 | 29 |
| 33 | Ni-Al42-Y1 | 3.4 | 30 |
| 34 | NiAl-0.05Y-0.05La | 3.5 | 29 |
| 35 | NiAl+1vol.%Y_2_O_3_ | 3.6 | 28 |
| 36 | NiAl+Al_2_O_3_ | 3.6 | 31 |
| 37 | Ni-Al49.9-Zr0.1 | 4.47 | 27 |
| 38 | Ni-31.44Al-0.21Hf | 4.56 | 32 |
| 39 | NiAl-0.05Hf-0.05La | 5.27 | 29 |
| 40 | NiAl | 5.8 | 31 |
| 41 | NiAl | 6.69 | 33 |
| 42 | Ni-31.44Al-0.21Hf (Preoxidized) | 7.63 | 32 |
| 43 | NiAl | 9 | 24 |
| 44 | Ni-Al25-Y0.1 | 9.62 | 34 |
| 45 | Ni-Al49.95-Hf0.05 | 10.07 | 35 |
| 46 | Ni-Al42-Ti1 | 10.22 | 30 |
| 47 | NiAl | 12 | 28 |
| 48 | NiAl+Ni-cotaedAl_2_O_3_ | 13.9 | 31 |
| 49 | NiAl+10vol.%Ti_2_B | 16.1 | 33 |
| 50 | NiAl | 19.5 | 29 |

**Table S2.** Chemical composition (at.%)of the γ-pheas in the MPEA and MCrAlY measured by EDS.

|  | Ni | Co | Cr | Al |
| --- | --- | --- | --- | --- |
| MPEA | 29.5 | 27.5 | 30 | 13 |
| MCrAlY | 35.8 | 26.6 | 25.8 | 11.8 |

**Table S3.** Aspect ratio of β-phase in MPEA and MCrAlY measured by ImageJ

|  | *l/r* |
| --- | --- |
| MPEA | 32.05 |
| MCrAlY | 2.36 |

**Table S4.** Chemical composition (at.%) and configurational entropy (*H*) and of the ADZ in the MPEA and MCrAlY.

|  | Chemical composition of the ADZ (at.%) | | | | configurational entropy (J/mol·K) |
| --- | --- | --- | --- | --- | --- |
|  | Ni | Co | Cr | Al |  |
| MPEA | 30.4 | 27.2 | 30.1 | 12.3 | 11.1 |
| MCrAlY | 40.3 | 27.2 | 25 | 7.5 | 10.5 |

**Table S5.** The effective interatomic distance (*deff i*) in each of constituent elements and the average interatomic distance ($\bar{d}$) in ADZ supercell structures of MPEA and MCrAlY

|  | *deff Ni* (Å) | *deff Co* (Å) | *deff Cr* (Å) | *deff Al* (Å) | $\bar{d}$ (Å) |
| --- | --- | --- | --- | --- | --- |
| MPEA | 2.9015 | 2.8705 | 2.8581 | 3.1281 | 2.9093 |
| MCrAlY | 2.8400 | 2.9066 | 2.8864 | 3.0703 | 2.8865 |

**References**

1. K. Ma, J. M. Schoenung, “Thermodynamic investigation into the equilibrium phases in the NiCoCrAl system at elevated temperatures,” *Surface and Coatings Technology* (2010): 205 (7), 2273. <https://doi.org/https://doi.org/10.1016/j.surfcoat.2010.09.009.>
2. N. P. Kubheka, S. F. Shange, S. C. Onwubu, N. Deenadayalu, P. S. Mdluli, T. H. Mokhothu, “ImageJ analysis for quantifying lead ion in environmental water using gold nanoparticles as a colorimetric probe,” *Journal of Molecular Liquids* (2025): 420, 126804. <https://doi.org/https://doi.org/10.1016/j.molliq.2024.126804.>
3. G. K. Nayak, A. Kretschmer, P. H. Mayrhofer, D. Holec, “On correlations between local chemistry, distortions and kinetics in high entropy nitrides: An ab initio study,” *Acta Materialia* (2023): 255, 118951. <https://doi.org/10.1016/j.actamat.2023.118951.>
4. C. Lee, Y. Chou, G. Kim, et al., “Lattice-Distortion-Enhanced Yield Strength in a Refractory High-Entropy Alloy,” *Advanced Materials* (2020): 32 (49), 2004029. <https://doi.org/10.1002/adma.202004029.>
5. J. W. Yeh, S. Y. Chang, Y. D. Hong, S. K. Chen, S. J. Lin, “Anomalous decrease in X-ray diffraction intensities of Cu–Ni–Al–Co–Cr–Fe–Si alloy systems with multi-principal elements,” *Materials Chemistry and Physics* (2007): 103 (1), 41. <https://doi.org/10.1016/j.matchemphys.2007.01.003.>
6. C. Lee, G. Song, M. C. Gao, et al., “Lattice distortion in a strong and ductile refractory high-entropy alloy,” *Acta Materialia* (2018): 160, 158. <https://doi.org/10.1016/j.actamat.2018.08.053.>
7. S. Zhao, “Defect properties in a VTaCrW equiatomic high entropy alloy (HEA) with the body centered cubic (bcc) structure,” *Journal of Materials Science & Technology* (2020): 44, 133. <https://doi.org/10.1016/j.jmst.2019.10.025.>
8. M. Mizuno, K. Sugita, H. Araki, “Defect energetics for diffusion in CrMnFeCoNi high-entropy alloy from first-principles calculations,” *Computational Materials Science* (2019): 170, 109163. <https://doi.org/10.1016/j.commatsci.2019.109163.>
9. A. Roy, P. Singh, G. Balasubramanian, D. D. Johnson, “Vacancy formation energies and migration barriers in multi-principal element alloys,” *Acta Materialia* (2022): 226, 117611. <https://doi.org/10.1016/j.actamat.2021.117611.>
10. M. Liu, L. Zhang, W. Chen, J. Xin, Y. Du, H. Xu, “Diffusivities and atomic mobilities in fcc_A1 Ni–X (X=Ge, Ti and V) alloys,” *Calphad* (2013): 41, 108. <https://doi.org/https://doi.org/10.1016/j.calphad.2013.02.005.>
11. R. Bouchet, R. Mevrel, “A numerical inverse method for calculating the interdiffusion coefficients along a diffusion path in ternary systems,” *Acta Materialia* (2002): 50 (19), 4887. <https://doi.org/https://doi.org/10.1016/S1359-6454(02)00358-0.>
12. L. Li, Y. Chen, A. Huang, et al., “A novel NiCoCrAlPt high-entropy alloy with superb oxidation resistance at 1200 °C,” *Corrosion Science* (2024): 228, 111819.. <https://doi.org/10.1016/j.corsci.2024.111819.>
13. A. Huang, L. Li, X. Liu, et al., “Superior oxidation resistance of a Y-Hf co-doped Al18Co30Cr10Fe10Ni32 eutectic high-entropy alloy at 1100–1300 °C,” *Journal of Alloys and Compounds* (2024): 992, 174597.. <https://doi.org/10.1016/j.jallcom.2024.174597.>
14. M. P. Taylor, H. E. Evans, “Formation of diffusion cells in LPPS MCrAlY coatings,” *Materials at High Temperatures* (2014): 20 (4), 461. <https://doi.org/10.1179/mht.2003.053.>
15. J. Lu, H. Zhang, L. Li, et al., “Y-Hf co-doped Al1.1CoCr0.8FeNi high-entropy alloy with excellent oxidation resistance and nanostructure stability at 1200°C,” *Scripta Materialia* (2021): 203, 114105. <https://doi.org/10.1016/j.scriptamat.2021.114105.>
16. C. Tang, H. Shi, A. Jianu, et al., “High-temperature oxidation of AlCrFeNi-(Mn or Co) high-entropy alloys: Effect of atmosphere and reactive element addition,” *Corrosion Science* (2021): 192, 114105. <https://doi.org/10.1016/j.corsci.2021.109809.>
17. S. Kim, C.-H. Lee, J. H. Jang, J. Moon, J. H. Kim, C. B. Bahn, “High-temperature steam oxidation of (Ti, Mo)C-forming FeCrAlY alloy,” *Journal of Nuclear Materials* (2023): 574, 154220. <https://doi.org/10.1016/j.jnucmat.2022.154220.>
18. B. A. Pint, A. J. Garratt-Reed, L. W. Hobbs, “The reactive element effect in commercial ODS FeCrAI alloys,” *Materials at High Temperatures* (2016): 13 (1), 3. <https://doi.org/10.1080/09603409.1995.11689496.>
19. B. A. Pint, “Optimization of Reactive‐Element Additions to Improve Oxidation Performance of Alumina‐Forming Alloys,” *Journal of the American Ceramic Society* (2004): 86 (4), 686. <https://doi.org/10.1111/j.1151-2916.2003.tb03358.x.>
20. C. Badini, F. Laurella, “Oxidation of FeCrAl alloy: influence of temperature and atmosphere on scale growth rate and mechanism,” *Surface & Coatings Technology* (2001): 135 (2-3), 291. <https://doi.org/10.1016/S0257-8972(00)00989-0.>
21. B. Karpe, J. Kovač, M. Manfreda, et al., “Element redistribution in the oxide scale during air oxidation of Kanthal AF alloy,” *Journal of Materials Research and Technology* (2024): 33, 8720. <https://doi.org/10.1016/j.jmrt.2024.11.169.>
22. Z. Yang, J. Pan, Z. Wang, et al., “New insights into the mechanism of yttrium changing high-temperature oxide growth of Fe-13Cr–6Al–2Mo–0.5Nb alloy for fuel cladding,” *Corrosion Science* (2020): 172, 108728. <https://doi.org/10.1016/j.corsci.2020.108728.>
23. W. Zhang, Y. Qian, R. Sun, et al., “Oxidation characteristics of Fe22Cr5Al3Mo-xNb alloys in high temperature steam,” *Corrosion Science* (2021): 191, 109722. <https://doi.org/10.1016/j.corsci.2021.109722.>
24. B. A. Pint, K. L. More, I. G. Wright, “Effect of quaternary additions on the oxidation behavior of Hf-doped NiAl,” *Oxidation of Metals* (2003): 59 (3-4), 257. <https://doi.org/10.1023/A:1023087926788.>
25. S. Jeong, D. Lee, “The effect of fe on high temperature oxidation of NiAl,” *METALS AND MATERIALS INTERNATIONAL* (1998): 4 (5), 1077. <https://doi.org/10.1007/BF03025979.>
26. J. Jing, J. He, H. Guo, “Impact of Si addition on high-temperature oxidation behavior of NiAlHf alloys,” *Journal of Materials Science & Technology* (2019): 35 (9), 2038. <https://doi.org/10.1016/j.jmst.2019.04.023.>
27. L. Wei, H. Peng, F. Jia, L. Zheng, S. Gong, H. Guo, “Cyclic oxidation behavior of Hf/Zr co-doped EB-PVD β-NiAl coatings at 1200 °C,” *Surface and Coatings Technology* (2015): 276, 721. <https://doi.org/10.1016/j.surfcoat.2015.05.039.>
28. B. A. Pint, L. W. Hobbs, “The oxidation behavior of Y2O3-dispersed β-NiAl,” *Oxidation of Metals* (2004): 61 (3-4), 273. <https://doi.org/10.1023/B:OXID.0000025335.62402.79.>
29. H. Guo, D. Li, L. Zheng, S. Gong, H. Xu, “Effect of co-doping of two reactive elements on alumina scale growth of β-NiAl at 1200°C,” *Corrosion Science* (2014): 88, 197. <https://doi.org/10.1016/j.corsci.2014.07.036.>
30. T. L. Barth, P. K. Weber, T. Liu, et al., “Grain boundary transport through thermally grown alumina scales on NiAl,” *Corrosion Science* (2022): 209, 110798.. <https://doi.org/10.1016/j.corsci.2022.110798.>
31. J. Doychak, J. A. Nesbitt, R. D. Noebe, R. R. Bowman, “Oxidation of Al2O3 Continuous Fiber-Reinforced Nial Composites,” *Oxidation of Metals* (1992): 38 (1-2), 45. <https://doi.org/10.1007/BF00665044.>
32. X. Li, J. Zou, Q. Shi, et al., “Effect of Al2O3 scales from pre-oxidation on the microstructural evolution and phase transition of NiAlHf coatings at 1200°C,” *Surface and Coatings Technology* (2022): 433, 128119. <https://doi.org/10.1016/j.surfcoat.2022.128119.>
33. B. A. Pregger, T. Kircher, A. Khan, “Oxidation Behavior of A Nial/Tib2 Intermetallic Composite,” Materials Science And Engineering A-Structural Materials Properties Microstructure And Processing (1992): 153 (1-2), 567. <https://doi.org/10.1016/0921-5093(92)90252-V.>
34. Y. Zhu, W. Qian, F. Dai, Y. Ye, Y. Hua, J. Cai, “Study of oxidation mechanisms of Ni-Al alloys with different phase structures at 1200 °C,” *Materials Today Communications* (2023): 35, 106393. <https://doi.org/10.1016/j.mtcomm.2023.106393.>
35. L. Liu, J. He, B. Zhou, D. Fan, H. Guo, “The importance of grain boundaries on the reactive elements effect in β-NiAlHf alloy,” *Materials Letters* (2023): 342, 134305. <https://doi.org/10.1016/j.matlet.2023.134305.>
